# KRAS Mutation Subtypes and Their Association with Other Driver Mutations in Oncogenic Pathways

**DOI:** 10.3390/cells13141221

**Published:** 2024-07-19

**Authors:** Koushik Mondal, Mahesh Kumar Posa, Revathi P. Shenoy, Susanta Roychoudhury

**Affiliations:** 1 Division of Basic & Translational Research, Saroj Gupta Cancer Centre & Research Institute, MG Road, Kolkata 700063, West Bengal, India; 2 Department of Cancer Immunology, SwasthyaNiketan Integrated Healthcare & Research Foundation, Koramangala, Bengaluru 560034, Karnataka, India; 3 School of Pharmaceutical Sciences, Jaipur National University, Jagatpura, Jaipur 302017, Rajasthan, India; posa.mahesh@gmail.com; 4 Department of Biochemistry, Kasturba Medical College, Manipal, Manipal Academy of Higher Education, Manipal 576104, Karnataka, India; revathi.shenoy@manipal.edu; 5 CSIR-Indian Institute of Chemical Biology, 4 Raja S.C.Mullick Road, Jadavpur, Kolkata 700032, West Bengal, India

**Keywords:** KRAS, mutation, cBioPortal, domain, signaling pathway, prognostic response, predictive response, therapeutic strategy

## Abstract

The KRAS mutation stands out as one of the most influential oncogenic mutations, which directly regulates the hallmark features of cancer and interacts with other cancer-causing driver mutations. However, there remains a lack of precise information on their cooccurrence with mutated variants of KRAS and any correlations between KRAS and other driver mutations. To enquire about this issue, we delved into cBioPortal, TCGA, UALCAN, and Uniport studies. We aimed to unravel the complexity of *KRAS* and its relationships with other driver mutations. We noticed that G12D and G12V are the prevalent mutated variants of KRAS and coexist with the TP53 mutation in PAAD and CRAD, while G12C and G12V coexist with LUAD. We also noticed similar observations in the case of PIK3CA and APC mutations in CRAD. At the transcript level, a positive correlation exists between *KRAS* and *PIK3CA* and between *APC* and *KRAS* in CRAD. The existence of the co-mutation of KRAS and other driver mutations could influence the signaling pathway in the neoplastic transformation. Moreover, it has immense prognostic and predictive implications, which could help in better therapeutic management to treat cancer.

## 1. Introduction

KRAS, part of the RAS family of oncoproteins, is known for its GTPase activity, which regulates various cell-signaling pathways. It is the most frequently mutated isoform in human cancers (85%), followed by NRAS (11%) and HRAS (3%) [1]. The human *KRAS* gene, located on 12p12.1, has six exons and two splice variants, with *KRAS4B* being highly expressed and *KRAS4A* weakly expressed [2], though recent studies have shown a widespread expression of *KRAS4A* in cancer [3]. Epithelial cancers are primarily affected by KRAS mutations, with approximately 90% of pancreatic adenocarcinomas (PAAD) associated with KRAS mutations, followed by colorectal carcinoma (45%) and lung adenocarcinoma (30%) [1]. The *KRAS* gene encodes two highly related protein isoforms, KRAS4B (188 aa) and KRAS4A (189 aa), with low molecular masses of approximately 21.6 kDa [4,5]. The active state of KRAS is achieved by a conformational change with the exchange of GTP for GDP [4,6]. This “on and off” switching of KRAS is regulated by guanine exchange factors (GEFs) and GTPase-activating proteins (GAPs), which, respectively, load GTP onto KRAS and hydrolyze it back to GDP [7,8]. Oncogenic KRAS mutations, especially at Codons G12, G13, and Q61, impair GTPase activity, leading to constitutive activation and aberrant signaling in cancer. Despite decades of research, therapeutic strategies against KRAS mutations remain challenging. Recent advancements, such as the use of sotorasib-targeting KRAS^G12C^ mutants in non-small cell lung carcinoma (NSCLC), have shown clinical benefits [9]. However, developing drugs against KRAS is hindered by its high affinity for GTP and the lack of pharmacologically accessible binding pockets for small molecule inhibitors in its catalytic domain [1,10,11].

## 2. KRAS Protein Domain, Structure, Function, and Pattern of Mutation

**Figure 1:**
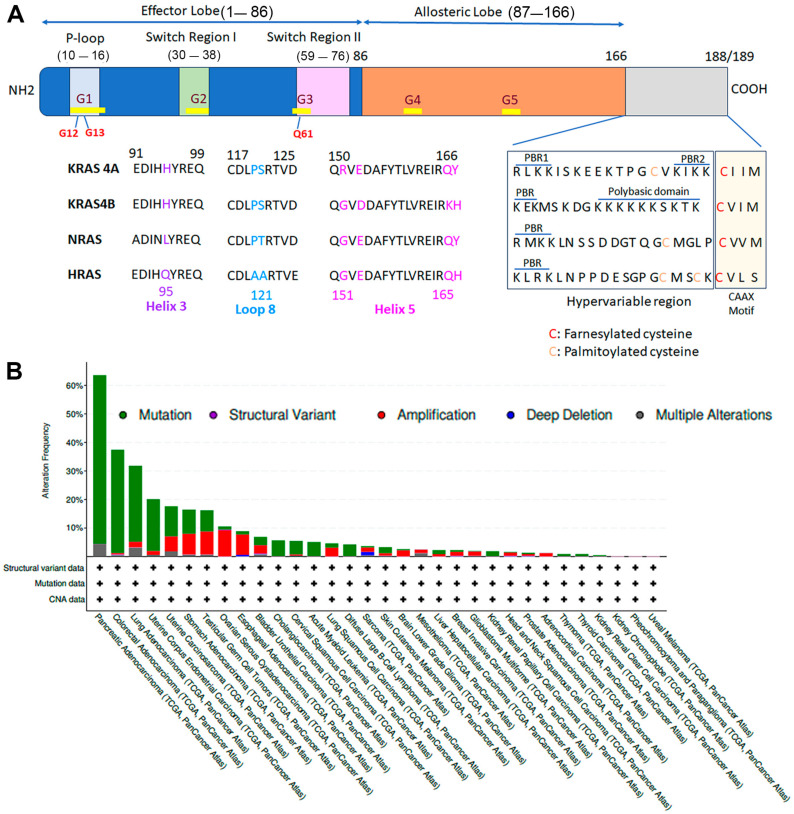
The structure of RAS proteins and prevalence of KRAS mutations in different types of cancer. (**A**) shows the structure of different isoforms of RAS proteins (KRAS4A, KRAS4B, NRAS, and HRAS). The areas represented with the blue box show the effector lobe (1–86 aa), the orange box as the allosteric lobe (87–166 aa), and the light gray box as a hypervariable region (167–188/189 aa). In the effector lobe, there are P-loop (10–16 aa), Switch Part I (30–38 aa), and Switch Region II (59–76 aa). Yellow boxes (G1–G5) refer to the conserved region responsible for the exchange of guanine nucleotide. The “hotspot” region (12, 13, and Q61) of KRAS remained at the G2 and G3 regions of the protein. The G-domain of RAS family proteins is highly conserved; however, the variability exists at Helix 3, Helix 5, and Loop 8. At Helix 3, Position 95, KRAS is highly conserved and has H (His) for both 4A and 4B, whereas NRAS and HRAS are substituted with L (Leu) and Q (Gln), respectively. In Loop 8, amino acid P (Pro) at Position 120 of KRAS and NRAS is replaced with A (Ala) for HRAS. In comparison, S (Ser) remained conserved at 121 for KRAS4A and 4B. However, S (Ser) is substituted with T (Thr) and A (Ala) in NRAS and HRAS, respectively, at the 121st position of the protein. In Helix 5, Amino Acid Positions 151, 153, 165, and 166 varied among the RAS isoforms. At 151, the R (Arg) of KRAS4A is replaced with G (Gly) of KRAS4B. KRAS4B, NRAS, and HRAS have conserved residues of G (Gly) at Amino Acid Position 151. Next, at 153, amino acid, E (Glu), is conserved among KRAS4A, NRAS, and HRAS, whereas KRAS4B has 153D (153Asp), instead of E (Glu). At Position 165, KRAS4A, NRAS, and HRAS have Q (Gln), whereas Q (Gln) is substituted with K (Lys) in the case of KRAS4B. Both KRAS4A and NRAS have Y (Tyr) at 166th position, while it is occupied with H (His) for KRAS4B and HRAS. There is a conserved motif of CAAX, where cysteine (Cys) is farnesylated, which is denoted as red. Immediately upstream of the CAAX motif in the hypervariable region, there is only about 10–15% homology among four RAS isoforms. In that region, orange is denoted as the site of palmitoylation of Cys residue. KRAS 4B is not palmitoylated; instead, it has a long stretch of lysine as a polybasic domain, while HRAS has two palmitoylated Cys residues. (**B**) shows the percentages of KRAS mutations in different types of cancer according to the cBioPortal database. TCGA pan-cancer information observed the highest level of KRAS mutation in pancreatic adenocarcinoma, followed by colorectal adenocarcinoma, lung adenocarcinoma, and uterine corpus endometrial carcinoma. Here, green denotes the missense mutations, purple is the structural variant, red is amplification, blue is deep deletion, and grey is multiple alterations.

The *RAS* gene family encodes four protein isoforms (KRAS4A, KRAS4B, HRAS, and NRAS), all of which are frequently mutated in various cancer types, including solid tumors and hematological cancers. A key distinguishing feature among these isoforms is their carboxy-terminal hypervariable region (HVR), with minimal variations in the G-domain [12]. The G-domain of RAS proteins (Residues 1–166) comprises an effector lobe (Residues 1–86) and an allosteric lobe (Residues 87–166) (Figure 1A). This domain is highly conserved among RAS isoforms, with 90% sequence homology, and features six beta-strands surrounded by five alpha-helices [13]. KRAS has four binding domains: the P-loop phosphate-binding loop (Residues 10–18 and Residues 56–59), a small molecule-binding pocket (Residues 29–35), and a fourth binding region (Residues 116–119) that interacts with the guanine base [14,15]. The core effector region (Residues 32–40) facilitates interactions with downstream effectors and GAPs. The functional domain in the effector lobe includes Switch Region I (Residues 30–38) and Switch Region II (Residues 59–76) (Figure 1A) [15], which interact with effector proteins like RAF (rapidly activated fibrosarcoma), PI3K (phosphoinositide-3 kinase), and Ral GEF (Ral guanine exchange factors) [15]. The allosteric lobe regulates intra-protein communication and connects the effector lobe with membrane-interacting residues [16,17]. The orientation of RAS protein interaction with the catalytic domain and the bilayer is regulated by Switch Regions SI and SII. Atomistic molecular dynamic studies have identified two structured states of RAS protein orientation, mediated by different helices and beta-strands [18]. The bilayer interaction with the catalytic domain is mediated by Helices 3 and 4 in Orientation 1 and Beta-strands 1–3 and Helix 2 in Orientation 2 [18]. These two orientations are tethered with the carboxy-terminal hypervariable region (HVR). The interaction of the KRAS protein with GTP/GDP and effector proteins is determined by the cooperation between the P-loop, Switch I, and Switch II [2,13]. Despite the high conservation (about 80%) of RAS protein isoforms, major differences are observed at the HVR [12], whereas the notable variation at the G-domain of RAS isoforms is observed at Helix 3, Loop 8, and Helix 5 (Figure 1A) [19,20]. 

Besides the variability at Positions 165 and 166 in Helix 8 [20], we also observed the variation of amino acids at Position 151 (Figure 1A). This evolutionary divergence among amino acids at the G-domain of RAS isoforms could influence the stringency of effector molecules’ interactions [21]. The C-terminus of RAS protein isoforms consists of a CAAX motif (C, cysteine; A, aliphatic amino acid; and X, any amino acid), and a bipartite polybasic region (PBR) (Figure 1A) [20,21]. Different types of posttranslational modification were reported at the C-terminal region of RAS proteins, where farnesylation and palmitoylation play a significant role in the membrane localization and anchoring of KRAS and other RAS isoforms [22]. The HVR domain (Residue 166–188/9) at the C-terminus plays a crucial role in membrane anchoring, which shows less than 15% of sequence homology among RAS proteins [20]. The conserved amino acid Cys in the CAAX motif is the site of farnesylation of RAS isoforms. The HVR of KRAS isoforms are highly positively charged compared to other RAS isoforms because of the abundance of lysine residues [20]. KRAS4B has a continuous stretch of lysine residues (PBR) and a farnesylation site at the CAAX motif (Figure 1A), while KRAS4A has two polybasic regions (PBR1 and PBR2) (Figure 1A), besides a site of palmitoylation at Cys180 [20]. The farnesylation site is conserved among all the RAS isoforms at the CAAX motif (Figure 1A) [20]. Like KRAS4A, NRAS has one palmitoyl and farnesyl site (Figure 1A) [20]. The site of palmitoylation occurs at Cys181 (Figure 1A). Unlike NRAS, the HRAS isoform has two palmitoyls and one farnesyl site, and the palmitoylation site occurs at Cys181 and Cys 184 (Figure 1A) [20]. Those conserved amino acids for posttranslational modification at HVR help RAS protein attach to the membrane. This was supported by the mutational substitution of respective amino acids or blocking posttranslational modification with inhibitors, leading to the failed localization of RAS isoforms at the membrane [3,20]. The altered amino acid residues at the HVR of different RAS isoforms could influence the functional redundancy of RAS proteins in cancer biology. Compared to KRAS4B, KRAS4A uses two motifs for trafficking towards the plasma membrane, where State 1 exists as a farnesylated form. In contrast, State II is farnesylated and palmitoylated, similar to NRAS [3]. Like KRAS4A, HRAS also exists in two states, and besides farnesylation, 181Cys- and 184Cys-mediated palmitoylation help membrane trafficking and anchoring [23]. Mono or di palmitoylation chooses the HRAS to be anchored with either the PM’s (Plasma membrane) cholesterol-rich or cholesterol-independent microdomain region [23]. This suggests that palmitoylation decides the HRAS’s interaction with a raft or non-raft PM [23]. Palmitoylation also regulates subcellular trafficking and the localization of HRAS and influences the downstream activation of RAF, MEK, and ERK [23]. In the case of NRAS and HRAS, the cycle of depalmitoylation and palmitoylation, golgi trapping, and involvement of secretory pathways helps in their trafficking and localization [24]. Palmitoylation helps to balance the entropy-driven changes in the cell of lipidated N- and H-RAS [24]. Interestingly, KRAS4B, which does not have the site of palmitoylation, counteracts the entropy-driven changes through the binding with cytosolic chaperone protein, δ-subunit of cGMP phosphodiesterase Type 6 (PDE6δ) [24], whereas KRAS4A balances the entropy-driven changes in the cell by palmitoylation, not through the binding of the cytosolic chaperone protein in the case of KRAS4B. Similar to N- and H-RAS, Golgi apparatus plays a key role in trafficking in the case of KRAS4A [3]. This could be the reason for the non-participation of PDE6δ with KRAS4A. The involvement of the Golgi apparatus with KRAS4B is minimal or absent in its membrane trafficking [25]. These changes in the RAS isoforms could have evolutionary significance in the cell and their differential expression in specific tissues [12]. Another important aspect of the RAS isoform-mediated-signaling event is its association with calmodulin (CaM), which promotes downstream signaling while interacting with KRAS [26]. The association of the CaM-mediated downstream signaling event is absent either in NRAS or HRAS [24]. Oncogenic KRAS4B specifically binds with CaM and sequesters farnesyl moiety from the membrane [27]. The allosteric interaction between CaM and KRAS4B leads to a conformational change in the RAS-binding domain (RBD), increasing the affinity towards PI3K-mediated downstream signaling compared to the RAF/MAPK pathway [28], whereas KRAS4A binds with CaM at State 1 (farnesylated) instead of State 2 (both farnesylated and palmitoylated) [20]. This characteristic could be useful for drug designing mediated by KRAS4A. Moreover, HVR can influence the state of orientation of RAS isoforms and the exchange of GTP/GDP. Reciprocately, the GTP/GDP exchange could also rearrange the orientation of the protein. It allows for the differential distribution of positive charge at the surface of the catalytic domain, leading to a differential pattern of engagement with the surface of the negatively charged bilayer [29]. This GTP-bound state of RAS occludes the effector-binding surface with the membrane [18]. The GTP-bound state also observes the minimal interaction of the HVR domain with the catalytic domain, which suggests that GDP loading leads to the catalytic domain’s interaction with the HVR and restricts KRAS activation of effector factors [30]. The sequence diversity among four RAS isoforms mainly resides at the allosteric lobe and the hypervariable region of the protein, which could lead to functional differences in RAS isoforms. Moreover, there are differences in the posttranslational modification among RAS isoforms, which could influence the pathogenicity of different mutations associated with RAS isoforms. The striking differences among RAS isoforms with their association with specific types of cancer suggest a more detailed understanding of RAS isoforms is necessary for their effective therapeutic management.

Among different RAS isotypes, KRAS is the prevalent mutation associated with different types of cancer, namely, pancreatic, colorectal, and lung carcinoma, while the incidence of NRAS and HRAS mutation is less among these three types of cancer. NRAS is prevalent in malignant melanoma and hematopoietic malignancy, while HRAS is observed mainly in bladder and cervical cancers [31]. According to the cBio Cancer Genomics Portal (cBioPortal), a pan-cancer study of The Cancer Genome Atlas (TCGA) on different types of cancer observed the highest percentages of KRAS mutation in pancreatic adenocarcinoma (PAAD) followed by colorectal adenocarcinoma (CRAD), lung adenocarcinoma (LUAD), and uterine corpus endometrial carcinoma (UCEC) (Figure 1B). Data availed from cBioPortal observed mutation in KRAS are mainly missense (Figure 1B). In the case of pancreatic cancer, the mutation of KRAS plays a critical role in cancer initiation and progression, which could be readily detectable at Stage 1 pancreatic intraepithelial neoplasia (PanIN) [32]. A study on pancreatic cancer reported that about 25% and 38% of KRAS mutations detected at the PanIN-1A and PanIN-1B stages, respectively [32]. This indicates the involvement of KRAS mutations in the early event of human pancreatic cancer. Similarly, in lung adenocarcinoma, the early lesion detected a mutation of KRAS [33]. Marabese et al., in their study on non-small cell lung carcinoma (NSCLC), detected the highest mutation rate of KRAS at diagnosis Stage IV, and the G3 grade observed about 74% of mutated KRAS [34]. In the case of colorectal cancer (CRC), though a significant percentage of patients have KRAS mutations, the primary initiating event of neoplasm is mediated by either the loss of function of APC or mutation of β-catenin [35]. In cancer cells, mutations in the KRAS gene are most frequently found at Positions 12 and 13 in exon one and less often in Codons 61, 63, 117, 119, and 146 [36]. The mutations are located near the site where GTP binds (Figure 1A). Table 1 informs different point mutations in pancreatic, colon, rectal, and lung adenocarcinoma and uterine endometrial carcinoma following the cBioPortal database. In PDAC, the prevalent alteration is observed at Codon 12. A Codon 12 allelic mutation results in glycine becoming aspartic acid, valine, and arginine, which observed the highest incidence of mutation at G12D (41.80%), followed by G12V (27.04%) and G12R (21.31%) (Table 1). The mutation at G12 occurs at the P-loop of the catalytic G-domain of KRAS (Figure 1A), the region that regulates the interaction between GTP/GDP and the effector protein [2,13]. In the murine model, the conditional endogenous expression of Kras^G12D^ in the embryonic fibroblasts stimulated the downstream pathways, proliferation, and neoplastic transformation [37]. In the case of colorectal carcinoma (CRC) patients, the most common mutations observed were G12D and G12V [38,39]. Recently, a small pool of Malaysian colorectal cancer patients (*n* = 33) observed the highest frequency of KRAS^G12D^ mutations, followed by G12V, G13D, and G12S [40]. Similar to PAAD, G12D mutation is the most common mutation in colon adenocarcinoma (26.57%), followed by G12V (20.27%) and G13D (16.78%) (Table 1), whereas rectal adenocarcinoma, G12V, has the highest frequency of occurrence (30%), followed by G12D (20%) and G13D (18%) (Table 1). 

The murine model of CRC revealed increased lymph node metastasis with Kras^G12V^ compared to Kras^G13D^ [41]. Moreover, the murine model of the Kras^G12V^ primary tumor had higher tumor cell survival and invasiveness, and C-X-C chemokine receptor 4 (CXCR4) overexpressed intravasated tumor emboli [41]. CXCR4 is one of the key agents to monitor the crosstalk between the tumor cell and tumor microenvironment and promotes tumor progression [42]. The study of the cBioportal dataset observed that LUAD has the highest level of G12C mutation (40.9%), followed by G12V (23.39%), G12D (11.69%), and G12A (9.94%) (Table 1). In a bi-transgenic mouse model, the expression of human Kras^G12C^ in a lung-specific tetracycline-inducible manner demonstrated lung hyperplasia and well-differentiated adenomas [43]. The mutation of G12C is one of the diagnostic markers among KRAS mutations of lung cancer patients with tobacco exposure [44]. Not only lung cancer, but pancreatic and colorectal cancer also strongly correlate with the patient’s smoking habits. However, pancreatic and colorectal cancer patients with smoking habits were less associated with G12C mutations [45,46]. This indicates tissue-specific differences that exist with KRAS mutations during exposure to tobacco mutagens. In the case of advanced CRC, G13D mutations showed beneficial effects with anti-EGFR cetuximab therapy [47]. The in vitro isogenic cell lines and mouse model analysis of G12V colorectal cells were insensitive to cetuximab [47]. Unlike CRC, cetuximab and its beneficial effect on G13D-mutated cancer patients over G12D mutants were not observed in NSCLC [48]. Like pancreatic, colon, and rectum adenocarcinoma, data from cBioPortal also noticed the highest incidence of G12D mutation (34.04%) in uterine endometrial carcinoma (Table 1). Different types of KRAS mutations, namely, G12A, G12S, Q61H, A146T, and K117N, are also associated with different types of cancer. Based on the cBioPortal study, Table 1 shows different types of KRAS mutations observed in different types of cancers.

Compared to KRAS, the most frequently observed mutations of HRAS at Codons 12, 13, and 61 are G12V, G13R, and Q61R, respectively [49]. NRAS observes the most frequent mutation of G12D, G13D, and Q61R, respectively, at Codons 12, 13, and 61 [49]. Recently, the discovery of a new murine model, *LSL-Kras*, showed tissue-selective neoplastic transformation to understand the tumor initiation, progression, and therapy response in *Kras^G12D^*, *Kras^G12C^*, *Kras^G12R^*, and *Kras^G13D^* mutant models [50]. In this study, the authors noticed that the colons of Kras^G12C^ and Kras^G12D^ displayed dramatic hyperplasia compared to Kras^G13D^ with moderate hyperplasia [50], whereas Kras^G12R^ closely resembled the tissue architecture of Kras^WT^. In the same study, the Kras^G12D^ mutant model observed an increased level of neoplasm-initiating capacities in the pancreas (acini–acinar to ductal metaplasia–pancreatic epithelial neoplasia) compared to the other mutations [50]. Moreover, in the mouse embryonic fibroblast (MEF), the expression level of Ras–GTP mutant proteins was higher than the wild-type KRAS. Kras^G12D^ showed the highest expression level among the mutated KRAS, while Kras^G13D^ was the lowest [50]. Smith et al., in their study on the cell line model, observed that G12V and G12D demonstrated increased colony-forming ability compared to other KRAS mutations [51]. Mutations at Codon 12 showed greater transforming potential than Codons 13 and 61, with the highest prevalence of G12V followed by G12D > G13D > Q61H [51]. Comparing the transform potentiality of the G12V mutation of KRAS, KRAS4A showed higher potentiality than KRAS4B [52]. This indicates the differential ability of neoplastic transformation of RAS isoforms. The in vivo study, using different *KRAS* mutants transformed in the NIH3T3 cells and implanted into the Nu/Nu Swiss mice, observed more aggressive tumorigenicity among the animals harboring the Kras^G12V^ transformants compared to the Kras^G12D^ [53]. The hotspot mutations (12, 13, and 61) of RAS proteins are centered around the nucleotide-binding site of the protein, which is obvious with the amino acid alteration could impact GTP binding or intrinsic GTP hydrolysis and GAP-mediated GTP hydrolysis [8]. The higher transforming potential of G12V could be because of its high GTP binding property studied in the human MCF10A isogenic cell line [54]. The same study reported the highest level of the GTP binding capacity of Q61H mutated KRAS, which was about 5-to-6-fold higher than the control cells [54]. G12C, G12V, and G13C mutants showed two-fold increased capacity of GTP binding compared to control cells [54]. Hunter et al., in their in vitro study of the biochemical and structural analysis of KRAS mutant proteins, categorized KRAS mutants based on their intrinsic GTPase activity and RAF affinity [8]. RAF kinase is the immediate direct effector of KRAS in the Mitogen-activated protein kinase (MAPK) pathway and directly interacts with the switch one domain of KRAS [55]. Mutations at G12, G13, and Q61 observed impairment of GAP-mediated GTP hydrolysis and elevated level of cellular Ras-GTP expression [8]. According to the intrinsic enzymatic activity, KRAS mutants were categorized into high (G12C, G12D, and G13D) and low (G12A, G12R, G12V, Q61H, and Q61L) GTPase activity [8]. The faster GDP/GTP exchangeability of G13D could influence aggressiveness in its neoplastic ability to harbor this mutation [8]. Based on the RAF affinity, mutants were classified as high (G12A, G12C, G13D, and Q61L) and low (G12R, G12V, and G12D) RAF affinity [8]. The same study group also observed that among KRAS mutations, G12A and Q61L mutants had increased GAP-stimulated GTP hydrolysis capacity [8]. Comparing the invasiveness and metastasis characteristics of neoplastic transformation of KRAS4A and KRAS4B [56], Voice et al. observed that KRAS4B^G12V^ showed accelerated migration in the COS-7 transfected cells [52]. However, the expression level was higher for KRAS4A^G12V^ in the transfected cell [52]. In another study in the MCF10A cell line model, overexpressed G12D, G12V, and G13D showed increased cell migration in the transduced cell [54]. However, mutated KRAS did not show increased migratory abilities compared to wild-type KRAS at their physiological level [54]. 

KRAS mutation also influences or impacts other factors in cancer pathology. Mice models of *Kras^G12D^* and *p53^−/−^* showed 100% penetrance and increased tumor size compared to *Kras^WT/WT^p53^+/+^* mice [57]. Tumors from Kras^G12D^ p53^−/−^ mice metastasize to the liver, spleen, and kidney compared to their Kras^WT/WT^p53^−/−^ and wild-type counterparts [57]. The Kras^G12D^-driven pancreatic mouse model also influences metastasis through the heparan sulfate proteoglycan, Glypican 1 (Gpc1), where the loss of *Gpc1* restricts mesenteric metastasis [58]. This suggests that mutated KRAS not only influences the RAS downstream effector pathway but also interacts with other factors and influences the neoplastic property of the cell. In this regard, earlier studies on the application of immunotherapy among KRAS-mutated variants opened new horizons about the interaction between RAS mutation and immune-oncology (IO) biomarkers [59]. Recently, Salem and his colleagues sought to understand the interaction of IO biomarkers [microsatellite instability-high (MSI-H)/mismatch repair deficit status, tumor mutational burden (TMB), and programmed death ligand 1 (PD-L1)] and KRAS variant (KRAS^G12C^) using the next-generation sequencing analysis in the retrospective study of different types of cancer [60]. In this study, they noticed that KRAS^G12C^ variants were associated with high TMB status compared to KRAS^non-G12C^. In addition, they observed that the high expression of PD-L1 was associated with KRAS^G12C^ compared to the KRAS^WT^ (53.96% vs. 41.50%, OR = 1.65) and KRAS^non-G12C^ (53.96% versus 40.40%, OR= 1.73), respectively [60]. Studying further, MSI-H was less frequently associated with KRAS^G12C^ variants compared to KRAS^WT^ (1.17% versus 1.92%, OR = 0.63) and KRAS^non-G12C^ (1.17% versus 2.86%, OR = 0.39) mutated tumors [60]. In this section, we noticed that different types of KRAS mutated variants associated with different types of cancer influence the pathogenicity and regulate other factors, namely tumor-suppressive factors, cell-cycle regulators, and immune-response factors. This indicates that besides addressing the KRAS mutation, other information is necessary in the clinical management of cancer.

## 3. KRAS-Signaling Pathway and Crosstalk between Driver Mutations

**Figure 2:**
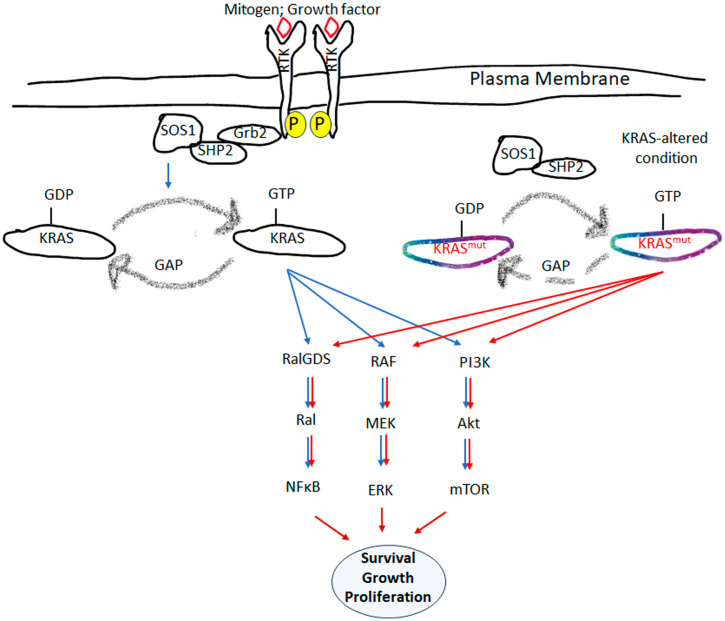
Activation of KRAS and downstream-signaling pathway. In normal physiological conditions, binding of growth factor to the plasma membrane-associated receptor tyrosine kinase (RTK) induces dimerization and phosphorylation of RTKs. Phosphorylated-RTKs eventually recruit the docking protein, growth factor receptor bound protein-2 (GRB2), and binding with the son of sevenless 1 (SOS1), which itself is a GEF. This activated SOS1, substituting GDP with GTP to KRAS, leading to subsequent conformational changes and activation of downstream factors. The transition from KRAS-GTP to KRAS-GDP, the inactive state, requires GTPase activating protein (GAP). In the activated form of KRAS, it targets downstream effector pathways, namely, (i) RAF/MEK/ERK, (ii) PI3K/Akt/mTOR, and (iii) Ral-GEF/Ral pathway. Mutated KRAS does not need the event of upstream activation. GEF-mediated activation state of KRAS^mut^ protein further activates the above-mentioned downstream effector pathways. Blue arrows denote normal physiological conditions, and red arrows indicate the KRAS-mutated state in cancer cells.

Though there is an extensive study on the KRAS-mediated-signaling pathway, a limited beneficial therapeutic outcome compels the necessity to understand KRAS and its upstream and downstream signaling factors, and their interactive partnership in designing the best therapeutic strategy. Also, we should consider different types of driver mutations and their association with the KRAS-signaling pathway. The signaling of the KRAS protein is mediated by the KRAS–GTP active state, which conveys the downstream signals through the respective effectors [61], where upstream and downstream factors regulate the RAS-mediated-signaling cascade. In the upstream event, upon binding with the ligand (namely, epidermal growth factor, fibroblast growth factor, and platelet-derived growth factor), the plasma membrane-associated receptor tyrosine kinase (RTK) induces an intermediary protein complex for further activation [62]. The immediate action of this interaction leads to the dimerization and phosphorylation of RTKs (epidermal growth factor receptor, fibroblast growth factor receptor, and platelet-derived growth factor receptor). Phosphorylated RTKs eventually recruit the docking protein, growth factor receptor-bound protein-2 (GRB2), and bind with the son of sevenless 1 (SOS1), which is itself a GEF [63] (Figure 2). This activated SOS1, substituting GDP with GTP to KRAS, leads to subsequent conformational changes and the activation of downstream factors (Figure 2).

Presently, we know that there are 56 bona fide RAS effectors with 12 functional classes with a “single effector-binding domain” [64,65]. Though it looked straightforward to target those effectors or their mode of function, an immense plasticity of interaction exists between the RAS-associated factors, making it a highly dynamic entity with enormous complexity. These dynamic associations regulate the competition of effector binding, differential levels of binding affinities and their abundances, and variable subcellular localization [66]. Interestingly, the oncogenic mutation of RAS need not be associated with the upstream activation events; rather, it activated constitutively as RAS-GTP and regulates the neoplasmic characteristics in the cell. Among all the RAS proteins, KRAS is the most malleable oncogenic mutation that influences the cell’s neoplastic transformation. The well-studied and characterized downstream effectors of KRAS are (i) RAF/MAPK/extracellular signal-related kinase (ERK), (ii) phosphatidylinositol 3-kinase (PI3K)/3-phosphoinositide-dependent protein kinase-1 (PDK1)/AKT, and (iii) RAS-selective guanine nucleotide exchange factors- RAS-like (RAL-GEF/RAL) pathway (Figure 2) [61]. All these pathways are controlled through the upstream ligand binding followed by GEF activation and effector function (Figure 2). While mutated, KRAS is constitutively activated with the GEF and forms the KRAS^mut^-GTP activated state, further instigating the effector pathway leading to neoplastic transformation (Figure 2). The specific mutations associated with KRAS regulate the specific effector molecule and its downstream signaling pathway. Previous studies informed that the mouse embryonic fibroblast of mutant Kras^G12D^ showed increased protein expression of phosphorylated-MEK, and ERK1/2 compared to the Kras^G12C^ and Kras^G13D^ mutations [50]. The authors further confirmed that all three mutations showed increased levels of phospho-AKT and S6. However, Kras^G12R^ had minimal influence on the RAS effectors [50]. In another study, a primary tumor from a bitransgenic *Kras^G12C^* mouse observed the phosphorylation of RAS-downstream effector factors: ERK, p38, p90 ribosomal S6 kinase (RSK), and MAP kinase-active protein kinase 2 (MAPKAPK-2) [43]. No activation of the c-Jun N-terminal kinase (JNK) and AKT pathways was noticed [43]. The experimental condition of Kras^G12V^ mutation observed that the primary tumor activated the downstream RAS-effector factor AKT, β5 integrin, and overexpression of vascular endothelial growth factor A (VEGFA) and serpin-1 [41]. In comparison, the mouse model of Kras^G13D^ tumors observed overexpression of integrin β1 and angiopoietin 2 [41]. The experimental model of the KRAS CRC model mimics the aggressiveness associated with G12V mutation, similar to the G12V mutated CRC patients [41]. In the NIH3T3 cell line, it was evidenced with RAF-dependent MEK phosphorylation pathway in the cells that have Kras^Q61L^ mutations, compared to Kras^G12V^mutations [67]. In contrast, in the mouse xenograft model, the higher growth rate of Kras^G12V^ tumors was associated with enhanced phosphorylation of retinoblastoma protein and upregulation of PCNA and Cyclin B, supporting faster G1/S and G2/M transitions [53]. The G12V mutated form of four RAS isoforms transfected in COS-1 cells observed the following hierarchy of RAF1 activation: KRAS4B > KRAS4A >>> NRAS > HRAS [52]. While in the NSCLC cell line, KRAS^G12D^ mutated cells followed the PI3K and MEK pathway compared to the KRAS^G12C^ and wild-type KRAS [68]. The preferential activation of the RAL pathway is regulated by hydrophobic G12C and G12V mutation compared to hydrophilic G12D mutation [68]. This observation points towards the dynamic ability of mutated KRAS to modulate effector pathways, which could be controlled through its intrinsic GTPase property, affinity towards RAF/PI3K factors, and their interactive partners [8]. Table 2 shows different types of signaling pathways associated with KRAS G12 mutations. Based on intrinsic GTPase activity and RAF affinity, Hunter et al., in their study, proposed a predictive model of the downstream pathway of KRAS mutations [8]. The study predicted that having high affinity with RAF kinase and lower rates of intrinsic hydrolytic activity, G12A, and Q61L mutated KRAS would preferentially signal through the RAF kinase pathway. In contrast, with a low affinity towards RAF and a faster hydrolysis rate, G12D mutations would not follow the RAF kinase-mediated downstream event [8]. In comparison, G12V and G12R, having slow intrinsic hydrolysis rates and low affinity towards RAF, would have a moderate activation of RAF kinase. Due to the higher affinity and intrinsic hydrolytic activity of G12C and G13D, an attenuated level of RAF kinase activity would be noticed compared to the G12A and Q61L mutations [8]. Here, we noticed that mutated KRAS variants influence different types of signaling pathways, so a thorough understanding of the signaling mechanism needs to be understood in in vitro and pre-clinical studies to implement the best therapeutic management in KRAS-mutated patients.

Numerous strategies exist to restrict those pathways and check tumor growth, proliferation, and survival. Unfortunately, no effective inhibitor could check the cancerous behavior of the cell. The unresponsiveness or resistance against those inhibitors is mainly due to the compensatory upregulation of different pathways and their cross-talk with other neoplastic factors [70,71]. The effector pathways (RAS-ERK and PI3K-MTOR) interact with each other and participate in tumorigenesis, which makes dual inhibitors comparatively effective toward therapeutic responses [72]. This cross-talk could depend upon the intensity or affinity of how the stimulus or ligands are induced or expressed, their cognate RTK association, and dependency upon various docking proteins [62]. In this regard, the example of agonists of the respective pathway will help to understand this puzzle. Where phorbol 12-myristate 13-acetate (PMA) is the strong activator of RAS-ERK and the weak activator of PI3K-MTOR [73], insulin and insulin growth factor-1 (IGF1) are the weak activators of RAS-ERK, but strong activators of PI3K-MTOR [74,75]. The complexity of this cross-talk could depend upon the stimulus’ property, intensity, and interacting partners. The downstream effectors of KRAS, the RAS-ERK, and PI3K-MTOR could cross-talk with each other either in a positive or negative feedback manner [76]. The components of the RAS–ERK pathway, namely RAS, RAF, ERK, and RSK, positively influence the PI3K–MTOR pathway. While TSC2 and mTORC1 act as the integration point to receive the inputs from RAS–ERK- and PI3K-signaling pathways [76]. Studies reported that hematopoietic cell lines FDC-P1 and TF-1 abrogate cytokine dependency while RAF1 is activated, the characteristic of tumorigenesis [77]. Though these cell lines did not show any abrogation on cytokine dependence while PI3K and AKT were activated, they positively affected cell survival. In comparison, in another hematopoietic cell line, FL5.12, the activation of PI3K and AKT synergizes RAF activation to abrogate cytokine dependency, which was not influenced by the RAF activation itself [77]. The same study reported an abnormal activation of RAS-ERK and PI3K-AKT while overexpressing HER2 in breast cancer [77]. Not only positively influencing each other, both have their negative feed onto the other, where the phosphorylation of ERK on Grb2-associated binding protein (GAB) and the phosphorylation of AKT on RAF negatively impact other pathways [78,79]. A cross-talk exists between KRAS mutation and MTOR hyperactivated mouse model in hepatocellular carcinoma (HCC), which acts through a paternally expressed-3 (PEG3)-mediated-signaling pathway [80]. The tumor tissue samples of transgenic *Kras^G12D^* mice observed a significant expression of PEG3 as a downstream target of KRAS/ERK/MTOR-driven HCC [80]. Further, it was observed that the cooperation of Kras^G12D^ and hyperactivation of MTOR markedly increased HCC formation along with lung metastasis [80]. The interaction between Kras^G12D^ and Tsc1 (Tuberous sclerosis complex 1) insufficiency-driven MTOR hyperactivation leads to the activation of the MEK/ERK/MTOR axis, not through the PI3K/AKT/MTOR axis [80]. The study also reported PEG3 as a novel factor of poor prognosis among Asian HCC patients with KRAS/ERK and MTOR hyperactivation, but not for non-Asian patients [80]. In NSCLC, the chemotherapy-resistance patients showed the activation of MTOR phosphorylation among KRAS-mutated variants (G12V and G13D), compared to the KRAS^WT^ patients [81]. This indicates a possible link between the KRAS and MTOR pathway.

Recently, Ibrahim and his colleagues used differential gene-expression analysis to understand its implication in cancer prediction or prognosis [82]. Their findings revealed that the mRNA level expression of the origin recognition complex 6 (ORC6) and S-phase kinase protein 2 (SKP2) acts as a promising predicting factor of breast cancer-specific survival (BCSS). In a multivariate Cox regression analysis, the combined high expression of *ORC6* and *SKP2* predicted shorter BCSS in the METABRIC cohort, Uppsala cohort, and Nottingham mRNA series [82]. These results were significantly associated with the prognostic factors, namely, tumor grade, lymph node status, and a well-known proliferation marker, Ki67 [82]. In another study, the correlation of mutation and mRNA expression of adenomatous polyposis coli (APC) and mutY homolog (MUTYH) was used to study the predisposition of hereditary colorectal polyposis [83]. Though two genes were positively correlated in cases and controls, controls showed stronger significance compared to the cases (Controls: Rho = 0.708, *p* < 0.001; Cases: Rho = 0.381, *p* = 0.02) [83]. The transcriptome measurement is easier than the proteome, where the abundance of mRNA is often used as a proxy of protein abundance. However, the integration of protein expression along with the mRNA level indicates valuable inference with its role in the pathology. The correlation of mRNA and protein in tumor proteomic profile often suffers with experimental reproducibility [84]. Kosti et al. integrated mass spectrometry data to understand a higher level of correlation between gene and protein expression in normal and cancer tissues [85]. It suggests a better bioinformatics tool is necessary to address the variability of mRNA–protein correlations. In the present article in search of a correlation between *KRAS* and other driver mutations, we studied cBioPortal analysis. To understand the correlation between *KRAS* and *MTOR*, we followed the mRNA correlation analysis following the cBioPortal. The mRNA correlation data analysis in cBioPortal provides information on whether the two genes are commonly upregulated or not. Moreover, through cBioPortal, we can identify the type of mutations associated with the coexisting mutations of two factors. Information from cBioPortal indicates that a negligible correlation exists between *KRAS* and *MTOR* in PAAD (Spearman: 0.21, Pearson: 0.16), CRAD (Spearman: −0.10, Pearson: −0.07), and LUAD (Spearman: 0.14, Pearson: 0.15) (Figure S1A–C). The mRNA expression data in the cBioPortal were calculated as a relative expression of a specific gene in a tumor sample to the gene’s expression distribution in a reference population of samples [86,87]. Total RNA transcript was determined by RSEM (RNA seq by Experimentation Maximization) and expression data from Illumina were batch-corrected to correct platform variations between GAII and HiSeq Illumina sequencers [87,88]. Though we did not notice positive correlation between *KRAS* and *MTOR* in PAAD, CRAD, and LUAD, in studying cBioPortal, we observed the existence of a co-mutation of these two factors in cancer. According to cBioPortal, the co-mutation of KRAS and MTOR noticed in PAAD are associated with G12D and G12V mutations of KRAS (Figure S1A). In the case of CRAD, the co-mutation of KRAS and MTOR are associated with the G12D, G12V, G13D, and A146T KRAS mutations, where a high prevalence of G12V and G13D KRAS mutations are associated with MTOR co-mutation (Figure S1B). In contrast, G12A and G12V KRAS mutations are mainly associated with MTOR mutations in LUAD (Figure S1C). In NSCLC, chemotherapy resistance is associated with mutated variants of KRAS G12V and G13D, where the MTOR pathway plays a significant role [81].

**Figure 3:**
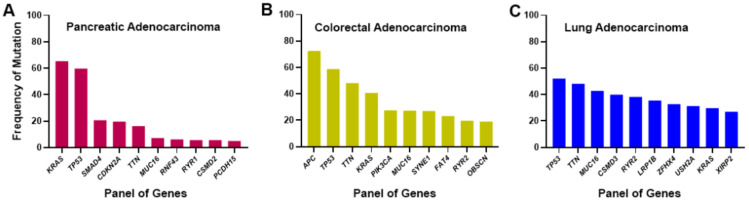
Panel of driver mutations in different types of cancer. Following the cBioPortal dataset, figures represented the first 10 driver mutation genes of pancreatic adenocarcinoma (PAAD) (**A**), colorectal adenocarcinoma (CRAD) (**B**), and lung adenocarcinoma (LUAD) (**C**). Data were collected from the cBioPortal of specific types of cancer and associated mutated gene frequencies of that cancer. The bar diagram represents the frequencies of the first 10 highly mutated genes in PAAD (**A**), CRAD (**B**), and LUAD (**C**). The abbreviated form of each gene has been mentioned as follows: *Kirsten Ras sarcoma virus* (*KRAS*) is the primary driver mutation gene associated with PAAD, followed by *Tumor protein 53 (TP53)*, *SMAD family member 4* (*SMAD4*), *Cylin-dependent kinase inhibitor 2A* (*CDKN2A*), Titin (TTN), *Mucin 16* (*MUC16*), *Ring finger protein 43* (*RNF 43*), *Rynodine receptor 1* (*RYR1*), *CUB, and Sushi multiple domain 2* (*CSMD2*), and *Protocadherin-related 15* (*PCDH15*). In the case of CRAD, *Adenomatous polyposis coli* (*APC*) is the primary driver mutation gene, followed by *TP53*, *TTN*, *KRAS*, *Phosphatidylinositol-4,5-bisphosphate 3-kinase catalytic subunit alpha* (*PIK3CA*), *MUC16*, *Spectrin repeat containing nuclear envelope protein 1* (*SYNE1*), *FAT atypical cadherin 4* (*FAT4*), *RYR2*, and *Obscurin* (*OBSCN*). While in LUAD, *TP53* is the primary driver mutation gene, followed by *TTN*, *MUC16*, *CSMD3*, *RYR2*, *LDL receptor-related protein 1B* (*LRP1B*), *Zinc finger homeobox 4* (*ZFHX4*), *Usherin* (*USH2A*), *KRAS*, and *Xin actin-binding repeat containing 2* (*XIRP2*).

This variability of the prevalence of different types of mutations in different cancers could be helpful in prognosis, which needs further study. Furthermore, coexisting different driver mutations should be taken into consideration in the treatment management of cancer, which has a significant impact on the therapeutic response. For example, colorectal cancer (CRC) patients with coexisting *KRAS* and *PIK3CA* (phosphatidylinositol 4,5-bisphosphate 3-kinase catalytic subunit alpha) mutations, do not respond to therapy [89]. The use of the MEK inhibitor alone, or PIK3CA inhibitor alone, did not provide a favorable response to this type of tumor [89]. PI3Ks are a group of lipid kinases that regulate cell signaling and are involved in cell proliferation, survival, adhesion, and motility. *PIK3CA* is among the first 10 driver mutations associated with CRAD (Figure 3B). Preclinical studies of *KRAS*- and *BRAF*-mutated CRC noticed the necessity of dual inhibition of MEK and PIK3CA pathways in the animal model of cancer [90]. Primary tumors of CRC having bi-mutations of *KRAS* and *PIK3CA* potentially develop liver metastasis and are associated with poor prognosis [91]. The study further reported that the higher the CRC staging, with Duke D-stage, patients had significantly elevated bimutations of *KRAS* and *PIK3CA* [91].

**Figure 4:**
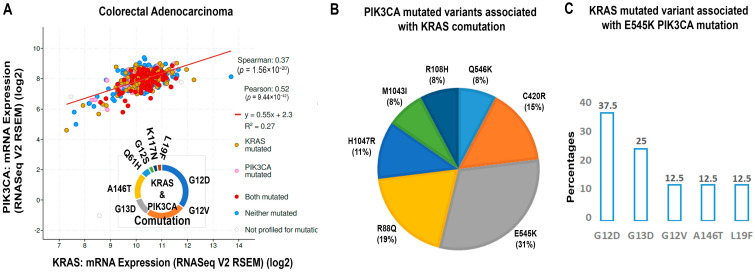
Mutant variants of KRAS with the coexisting mutations of PIK3CA in colorectal adenocarcinoma (CRAD). In the dot plot (**A**), mutated *KRAS* is presented as orange, mutated *PIK3CA* is presented as pink, the mutations of both *KRAS* and *PIK3CA* are presented as red, no mutation is presented as blue, and those not profiled for modification are presented as white. The KRAS-mutated variants are represented as a Doughnut diagram associated with the PIK3CA co-mutation. The high prevalence of mutations of G12D and G12V KRAS is concomitantly associated with PIK3CA mutation in CRAD (**A**). A positive correlation exists at the mRNA level between the PIK3CA and KRAS (Spearman’s coefficient: 0.37; Pearson’s coefficient: 0.52) (**A**). The pie diagram represents the first five prevalent PIK3CA mutations coexisting with mutated KRAS variants (**B**). Among the PIK3CA mutated variants, E545K represents the highest occurrence of co-mutation associated with mutated KRAS, followed by R88Q, C420R, and H1047R (**B**). Column diagram represents the percentages of KRAS-mutated variants associated with the E545K co-mutation of PIK3CA (**C**), where G12D remains the prevalent mutation, followed by G13D and others (G12V, A146T, and L19F) (**C**). Data collected from cBioPortal.

Similarly, in another study, CRC patients with concomitant mutations of *KRAS* and *PIK3CA* lead to poor clinicopathological parameters, including the location of the tumor at the proximal colon, the poorly differentiated state of the tumor, and significantly elevated levels of CA-199 and CA125 [81]. Scheffler et al., in their study, noticed that G12D was the most frequent *KRAS* mutation associated with *PIK3CA* co-mutation in non-small cell lung carcinoma (NSCLC) [92]. Further, studying cBioPortal, we noticed the co-mutation of *KRAS* and *PIK3CA* in CRAD (Figure 4A). In Figure 4A, the red color denotes the co-mutation of both *KRAS* and *PIK3CA*. We also observed the higher prevalence of G12D and G12V KRAS mutations, concomitantly associated with PIK3CA mutations (Figure 4A). Studying further, we observed that the E545K PIK3CA mutated variant is the prevalent co-mutated variant with KRAS mutation. Among the first five mutated variants of PIK3CA, E545K is the highest (37%) co-mutation associated with KRAS mutation, followed by R88Q (19%), C420R (15%), H1047R (11%), and 8% each of M1043I, R108H, and Q546K (Figure 4B). In addition, we observed that KRAS G12D is the prevalent (37.5%) mutation, followed by G13D (25%) associated with the co-mutation of the PIK3CA E545K variant (Figure 4C). Next, the cBioPortal study observed a moderate positive correlation (Pearson coefficient: 0.52) with linear regression between the mRNA expression of KRAS and PIK3CA (Figure 4A). The value of the coefficient of determination (R^2^ = 0.27) indicates that CRAD patients (27% of variability) are impacted by KRAS and PIK3CA (Figure 4A).

As reported by Li and colleagues, the bi-mutation of *KRAS* and *PIK3CA* leads to worse clinicopathological stages in CRC [91]. In the preclinical tumor cell line model, Nicolantonio and colleagues observed drug insensitivity in the co-mutation of *PIK3CA* and *KRAS* [93]. They observed that immortalized cancer epithelial cells that have hotspot PIK3CA mutation (H1047R or E545K) were sensitive to the MTOR inhibitor, rapamycin, and its analog everolimus. However, exogenously introduced *KRAS G13D* in the *PIK3CA*-mutated cancer cell leads to drug resistance [93]. Further translating this information for the identification of predictive therapeutic markers, the result showed similar outcomes to the preclinical result among a small group of cancer patients [93]. For further confirmation, this study needs to be repeated by recruiting more patients. Observing the information of Nicolantonio et al., Mohseni and Park predicted that concurrent *PIK3CA* mutation with the presence of mutation either in *KRAS* or *BRAF* could be resistant against everolimus, though there is the activation of the PI3K/AKT/MTOR pathway [93,94]. This suggests there could be the existence of an altered signaling pathway in this mechanism that requires advanced study. This alternative pathway could have existed because Hobbs et al., in their study, observed functionally distinct roles mediated by different signaling pathways controlled by different KRAS^mut^ variants [95]. In pancreatic cancer, KRAS^G12D/V^ regulates macropinocytosis but not KRAS^G12R^ [95]. This is because there is a defective interaction between KRAS^G12R^ and key effector PI3Kα, which is due to the structural perturbation in Switch II of KRAS^G12R^. The study further noticed a defective PI3K–AKT pathway and upregulation of MYC associated with KRAS^G12R^ mutation [95].

Though there is a co-existence of KRAS mutation and other RAS-effector mutations in the same tumor, *KRAS* and *BRAF* mutations do not occur in the same tumor. Nevertheless, both of them are within the same pathway [96]. For example, *KRAS* and *PIK3CA* mutations exist within the same tumor; conversely, *KRAS* and *BRAF* mutations are mutually exclusive [96,97]. This suggests that they may have similar functions. An earlier study observed that while BRAF was associated with microsatellite instability (MSI) in CRC, MSI was not associated with KRAS [96]. Contradicting earlier information that both of these factors are mutually exclusive, the dataset from cBioPortal shows that CRAD has a co-mutation of KRAS and BRAF (Figure S2B). In NSCLC, Q61X is the most prevalent mutation associated with the BRAF mutation mentioned by Scheffler and his colleagues [92]. Several studies reported a differential biological effect of KRAS versus BRAF oncogenes. To understand the transforming capability of *BRAF* and *KRAS* mutations, a colon carcinoma cell line was transfected with the *BRAF^V600E^* and *KRAS^G12V^* oncogenes, which observed that the mutation of *BRAF^V600E^* had the greater potential of a neoplastic effect than the mutated *KRAS^G12V^* [98], whereas mutated *KRAS* influences more efficient downstream ERK phosphorylation than the mutated *BRAF* [98]. In addition, mutated BRAF influences mRNA expression of the transcription factor and hypoxia-inducible factor-1 alpha (HIF-1α) and HIF-2α in normoxic conditions, while mutated KRAS did not modulate hypoxic factor in normoxia or hypoxic situations [99]. The level of HIF-1α mRNA remained unchanged while the KRAS mutation with siRNA was depleted, whereas mRNA level of both HIF-1α and HIF-2α were significantly reduced in BRAF knockdown cells. Further, the use of proteasome inhibitors confirmed the translational level regulation of HIF proteins mediated by KRAS and BRAF, where mutant KRAS regulated HIF1α translation through the PI3K pathway [99] and BRAF translated HIF2α through the MAPK pathway [99]. The use of PI3K and MAPK inhibitors further confirms the involvement of specific pathways regulated by KRAS and BRAF. On the other hand, it was reported that both *KRAS* and *BRAF* oncogenic mutations upregulated c-myc, promoting hyperproliferation and disrupting cell morphology [100]. Similarly, both the gain-of-function mutation in *KRAS* and *BRAF* work in synchronization and promote the expression of dual specificity phosphatase 4 (DUSP4) in a MEK-dependent manner, which negatively acts as a feedback mechanism to restrict nuclear localization of ERK1/2 in intestinal tumorigenesis [101]. Another interesting observation is the upregulation of the cancer stem cell marker, a cluster of Differentiation 133 (CD133), which determines the poor overall survival in colorectal cancer (CRC) and is regulated by the mutation of KRAS or BRAF [102]. Studies confirmed that patients with increased expression levels of CD133 in the tumor predicted poor relapse-free survival compared to the low CD133-expressed patients [102]. This suggests KRAS and BRAF could have similarities but also present differential oncogenic effects. This dynamic interaction could help to predict a model system for better prognosis and therapeutic response and the implementation of treatment management [103].

**Figure 5:**
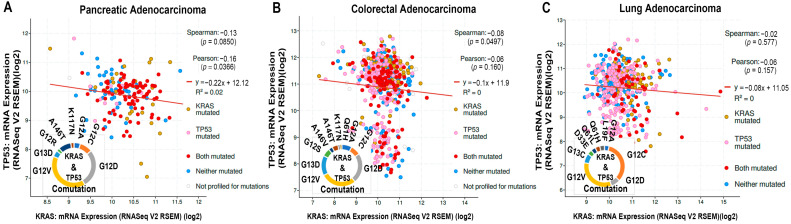
Mutant variants of KRAS with the coexisting mutations of TP53 in pancreatic adenocarcinoma (PAAD) (**A**), colorectal adenocarcinoma (CRAD (**B**), and lung adenocarcinoma (**C**). In the dot plots (**A**–**C**), mutated *KRAS* is presented as orange, mutated *TP53* is presented as pink, mutation of both *KRAS* and *TP53* are presented as red, no mutation is presented as blue, and not profiled for modification is presented as white. The KRAS-mutated variants are represented as a Doughnut diagram associated with TP53 co-mutation (**A**–**C**). The high prevalence of mutations of G12D and G12V KRAS is concomitantly associated with TP53 mutations in PAAD (**A**) and CRAD (**B**). In contrast, G12V and G12C KRAS mutations are mainly associated with TP53 co-mutation in LUAD (**C**). Though co-mutation exists, no significant correlation was observed between KRAS and TP53 at the level of mRNA expression (**A**–**C**). Data collected from cBioPortal.

The driver mutations differ in different types of cancer. It is well known that PAAD has the highest *KRAS* missense mutations, followed by colorectal and lung adenocarcinoma. Besides *KRAS*, *TP53* mutation is prevalently associated with many cancers. It is the gain of function of TP53, which exhibits the tumorigenic effect [104]. *KRAS*, *TP53*, and *MUC16* are among the first 10 driver mutations associated with PAAD, CRAD, and LUAD (Figure 3). Based on TCGA pan-cancer data, the percentages of co-mutation of *KRAS* and *TP53* remained highest in PAAD (54%), followed by rectal (31%) and colon (26%) adenocarcinoma and uterine carcinosarcoma (12%) [105]. In PDAC, the tumor suppressor TP53 predominantly undergoes a gain-of-function mutation along with KRAS. At the same time, the other factors, namely, SMAD Family Member 4 (SMAD4) and cyclin-dependent kinase inhibitor 2A (CDKN2A), lead to loss of protein expression [106]. Similarly, in cBioPortal, we noticed that *KRAS*, *TP53*, *SMAD4*, and *CDKN2A* are among the first four frequently mutated factors of PAAD (Figure 3A). Information collected from cBioPortal showed frequent co-mutation of *KRAS* and *TP53* in PAAD, CRAD, and LUAD (Figure 5A–C). Here, we consider only the gain-of-function mutation of TP53, which is the most prevalent type of TP53 mutation. Following the cBioPortal study, we noticed that G12D and G12V are prevalent KRAS mutations associated with TP53 co-mutation in PAAD and CRAD (Figure 5A,B). In LUAD, G12C and G12V are the prevalent mutations of KRAS with the TP53 co-mutation (Figure 5C). Though there is co-mutation, there is no clear inference on the correlation of mRNA expression between KRAS and TP53 (Figure 5A–C).

**Figure 6:**
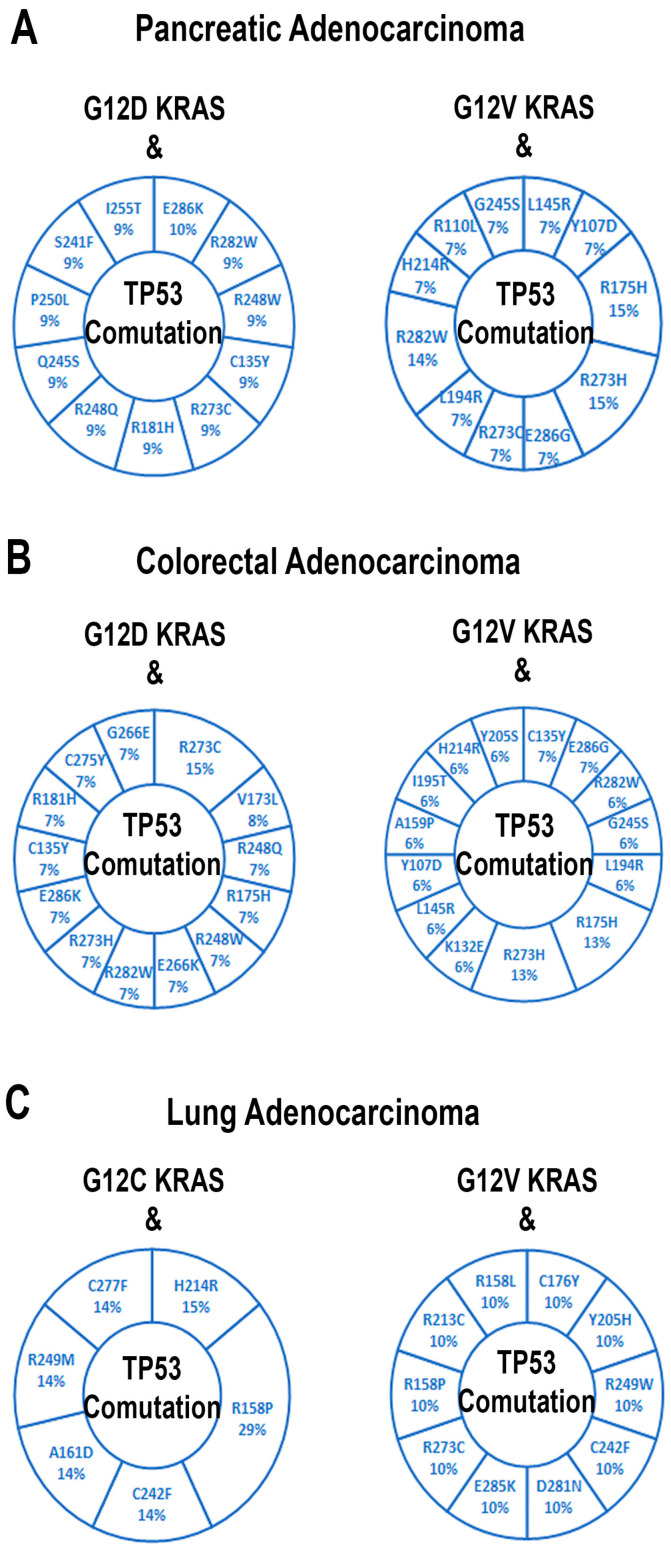
Co-mutation of TP53 associated with the prevalent KRAS mutations in different types of cancer. Prevalent mutations of KRAS (G12D and G12V) are associated with the missense mutations of TP53 in pancreatic adenocarcinoma (PAAD) (**A**). R175H, R273H, and R282W are the most frequent mutations of TP53 associated with G12V KRAS in PAAD. Prevalent mutations of KRAS (G12V and G12D) associated with the missense mutations of TP53 in colorectal adenocarcinoma (CRAD) (**B**). R175H and R273H are the most frequent mutations of TP53 associated with G12V KRAS and R273C is associated with G12D KRAS in CRAD. Prevalent mutations of KRAS (G12V and G12C) are associated with the missense mutations of TP53 in lung adenocarcinoma (LUAD) (**C**).

Further, studying cBioPortal, we observed different gain-of-function mutations of TP53 associated with G12D and G12V KRAS in PAAD and CRAD (Figure 6A,B). The prevalent mutations associated with G12V KRAS in PAAD are R175H, R273H, and R282W co-mutations of TP53 (Figure 6A), while R175H and R273H mutations are prevalent with G12V KRAS co-mutation in the case of CRAD (Figure 6B). Figure 6C showed different TP53 mutations associated with the co-mutation of G12C and G12V KRAS in LUAD. Studying cBioportal, we also noticed that the G12D KRAS mutation prevalently coexisted with the deletion mutant of TP53 in PAAD and CRAD.

In NSCLC, next-generation sequencing data showed *TP53* was the highest occurring co-mutation associated with *KRAS*, where G12A was the frequent variant associated with TP53 mutation [92]. In addition to the *TP53* co-mutation, this study further observed other driver mutations co-mutated with specific mutant variants of *KRAS* [92]. The co-mutation of different driver mutations could either blast the pathological expression of an essential target factor or costimulate varied types of neoplastic markers and induce pathological transformation. For example, in the experimental murine model study of *KRAS* oncogene and tumor-specific *TP53* missense mutation, oncogenic KRAS-driven cAMP responsive element-binding protein 1 (CREB1) phosphorylation by the MAPK/MEK pathway increased forkhead box protein A1 (FOXA1) upregulation and β-catenin stabilization, leading to the development of metastatic PDAC phenotypes [106]. In this study, Kim et al. showed that binding of mutated TP53 protein with the activated CREB1 augmented FOXA1 upregulation [106]. There was a significant elevation of FOXA1 and aberrant activation of β-catenin [106]. Pancreatic cancer patients with altered KRAS and TP53 had worse survival and compromised immune signatures [105]. Further, the study showed granulocyte-derived inflammasome activation and TNF signaling a putative mechanism of altered intra-tumor immune response and progenitor-like stemness properties [105]. In the in vitro study, chemoresistance was observed in the pancreatic epithelial cell line, transiently overexpressed with *KRAS^G12D^* and *TP53^R175H^* mutations [105]. In another study in PDAC, McIntyre and colleagues reported worse outcomes after a resection for the patients having alterations of both *KRAS* and *TP53* [107]. Studies on intrahepatic cholangiocarcinoma observed poor prognosis and significantly high tumor mutational burden among the patients having a co-mutation of *KRAS* and *TP53* compared to the wild type and single-mutation-harboring patients [108]. In the case of LUAD, patients with co-occurring *KRAS*/*TP53* mutations noticed remarkable clinical benefits while programmed death ligand-1 (PD-L1) blockade immunotherapy [109]. This variability, beneficial or detrimental, effect of coexisting mutations of *KRAS* and *TP53* differs from cancer to cancer and specific types of alterations. Targeting the pathway will not help in better outcomes to tackle the menace of KRAS mutations, but other factors also need to be considered.

**Figure 7:**
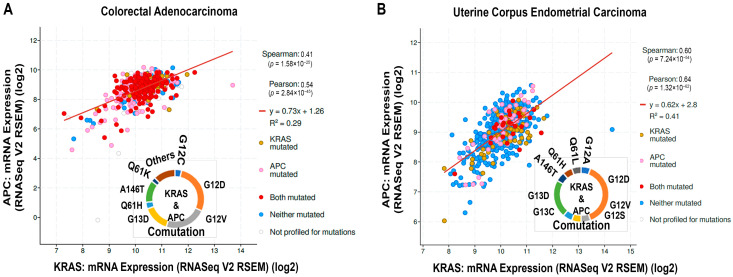
Mutant variants of KRAS with the coexisting mutations of APC in colorectal adenocarcinoma (CRAD (**A**), and Uterine Corpus Endometrial Carcinoma (UCEC) (**B**). In the dot plots (**A**,**B**), mutated *KRAS* is presented as orange, mutated *APC* is presented as pink, mutation of both *KRAS* and *APC* are presented as red, no mutation is presented as blue, and not profiled for modification is presented as white (**A**,**B**). The KRAS-mutated variants associated with APC co-mutation are represented as a Doughnut diagram (**A**,**B**). The prevalent KRAS mutations are G12D, G12V, and G13D, which are associated with APC co-mutation in CRAD (**A**), whereas G12D and G13D are prevalent KRAS mutations associated with APC co-mutation in UCEC (**B**). Dot plots show the positive correlation of *KRAS* and *APC* in CRAD (Spearman’s coefficient: 0.41; Pearson’s coefficient: 0.54) (**A**), and UCEC (Spearman’s coefficient: 0.60, Pearson’s coefficient: 0.64) (**B**). Data collected from cBioPortal.

Another common cancer-driving mechanism is the Wnt/β-catenin pathway that plays a crucial role in cancer. The aberrant modulation of the factors in this pathway leads to cell proliferation and transformation [110]. The gatekeeper of this pathway is Adenomatous polyposis coli (APC), which regulates the Wnt ligand-mediated signaling cascade and maintains cellular homeostasis [111]. Besides APC, other associated factors of regulatory protein complexes in the Wnt signaling cascade consist of Axin, casein kinase 1 (CK1), glycogen synthase kinase 3β (GSK3β), β-transducin repeat-containing protein (β-TrCP), lipoprotein receptor-related protein 5/6 (LRP5/6), Frizzled (FZD), disheveled (DVL), and β-catenin [112]. Though there is not enough space in the present context to discuss those factors of the Wnt signaling pathway, our focus is mainly on the cross-talk between KRAS and the Wnt pathway driver factor and regulation of cancer. Data from the cBioPortal informed that *APC* mutation contributes about 70%, while the involvement of *KRAS* is about 40% in CRAD (Figure 3B). Aberrant modification and cross-talk of these two pathways aggravate colorectal cancer initiation, progression, and metastasis [112]. The mutation associated with *APC* is mainly the loss of function mutation [112]. In cross-talk between the Wnt and RAS pathways, the loss of APC could influence the stability of the β-catenin and RAS protein [113]. What happens when the Wnt ligand is off? The beta-catenin destruction complex (βCdC) consisting of APC, axin, GSK3β, and CK1 phosphorylates β-catenin with the help of GSK3β. This same protein complex (βCdC) also phosphorylates RAS protein by GSK3β. Next, phosphorylated β-catenin and RAS protein eventually leads to proteasomal degradation associated with the E3 ubiquitin ligase protein, β-TrCP (Figure S3A). In the presence of Wnt ligands, this βCdC dissociates itself from the degradation mechanism of β-catenin and RAS proteins and is associated with the plasma membrane-bound complex LRP5/6, FZD, and DVL. Freed β-catenin eventually enters the nucleus and influences various neoplasmic factors (Figure S3B). The initiation and progression of CRC are mediated by the truncated APC, leading to a gain of function, instead of its tumor-suppressive action, leading to activation of the Wnt-signaling pathway and aberrant modulation of other factors [114]. Truncated APC initiates the hyperproliferation of epithelium and initiates dysplasia or early/intermediate adenoma. Eventually, other factors play their role, leading to the late adenoma [114]. The RAS protein is associated with the plasma membrane and further influences the downstream effector proteins. Though the individual mutation of APC and KRAS influences tumor growth and survival, the severity of CRC significantly rises when there are both mutations in the cell [115,116]. The murine model noticed liver metastasis with mutations in both *APC* and *KRAS* [113]. Information from cBioPortal conveys that co-mutation exists with the *KRAS*-mutated variants and the truncated mutants of *APC* in PAAD and UCEC (Figure 7). The prevalent KRAS mutations are G12D, G12V, and G13D, which are associated with the co-mutation of APC in the case of CRAD (Figure 7A). Meanwhile, G12D and G13D mutations are prevalently associated with the APC bi-mutations in UCEC (Figure 7B). Studying the cBioPortal, we noticed a positive correlation and linear regression between *KRAS* and *APC* (Spearman’s coefficient: 0.41, Pearson’s coefficient: 0.54) in CRAD (Figure 7A). Similarly, in UCEC, a positive correlation was noticed between *KRAS* and *APC* (Spearman’s coefficient: 0.60; Pearson’s coefficient: 0.64) (Figure 7B).

**Figure 8:**
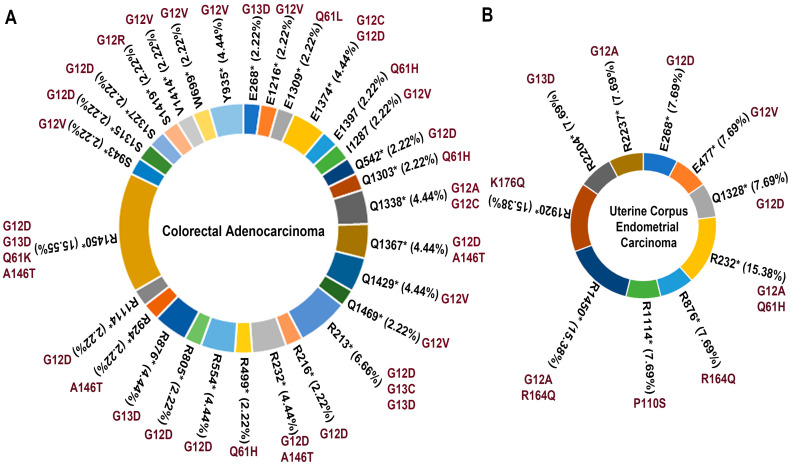
Mutant variants of APC with the coexisting mutations of KRAS in colorectal adenocarcinoma (CRAD) and Uterine corpus endometrial carcinoma (UCEC). Doughnut diagram (**A**,**B**) represents the APC-mutated variants associated with KRAS co-mutation. According to cBioPortal, R1450* represents the prevalent APC mutation associated with KRAS co-mutation in CRAD, followed by R213*, E1374*, Q1338*, R232*, and others (**A**). Along with the APC mutation, the associated co-mutation of KRAS mentioned in (A) in the case of CRAD. The prevalent APC mutations associated with KRAS co-mutation in UCEC are R232*, R1450*, and R1920* (**B**). Along with the APC mutation, the associated co-mutation of KRAS is mentioned in (**B**) in the case of UCEC.

Studying further, we observed that R1450* represents the prevalent APC mutation associated with KRAS co-mutation in CRAD, followed by R213*, E1374*, Q1338*, R232*, and others (Figure 8A), whereas, the prevalent APC mutations associated with KRAS co-mutation in UCEC are R232*, R1450*, and R1920* (Figure 8B). 

As neoplasmic transformation is a complex mechanism, we cannot ignore the involvement of other factors in this mechanism. We have noticed that a dynamic interactive mechanism exists in this cross-talk between the factors of different pathways and between different driver mutations. Also, the mutation in the same pathway could influence pathologic transitions or its beneficial effect. It has been reported that stimulation with Wnt3a activates the RAF1–MEK–ERK pathway [117]. In the murine model of both the targeted loss-of-function mutation of the *Apc* gene, *Apc*^1638N^, and a transgene encoding for the activated form of the human KRAS oncogene, the *pVillin-KRAS^G12V^* showed synergistic activation of two neoplasmic mutations, leading to severe neoplastic transformation [118]. In this experimental condition, APC mutation did not influence the AKT pathway; however, KRAS^G12V^ regulated the MAPK effector pathway [118]. Moreover, the mutation of KRAS^G12V^ induces phosphorylation of β-catenin at tyrosine residue, influencing the β-catenin pool towards the nucleus and increasing Wnt signaling [19]. The tyrosine phosphorylation of β-catenin regulates the release of E-cadherin from the cell’s tight junction and influences invasiveness in malignancy [118,119]. CRC cell lines with truncated APC mutation and constitutively activated KRAS protein induced the activation of the MAPK pathway and elevated level of Myc [120]. A selective use of specific inhibitors of individual pathways showed a poorer response than the combined superior outcome [73]. Most significantly, a combination of Wnt and KRAS pathway inhibitors downregulates CD44, which indicates CD44 as a target factor of a combined Wnt/KRAS signaling [120]. CD44 is the transmembrane adhesion molecule that acts as a hyaluronan receptor, a crucial component of the tumor extracellular matrix [121]. Studies revealed that frizzled coreceptor LRP6 is the probable convergence factor of the Wnt and KRAS pathway, where LRP6 is phosphorylated in an ERK1/2-dependent manner leading to oncogenesis [111]. A recent advancement in the research studies on CRC indicates cancer stem cell (CSC) plays a significant role in tumor development, progression, and metastasis, where a significant elevation of regenerating family member 4 (REG4) protein was noticed in the tumor samples of CRC patients [122]. In this mechanism, mutant KRAS plays a key driving force in enhancing CSC generation, which is further influenced by the APC mutations for a more severe malignant transformation of CRC and liver metastasis [122]. The incidence of co-mutation of different driver mutations could be the reason for complexity, hindering the prognosis and therapeutic management, as well. In human rectal cancer (RC), using a targeted sequencing approach, it was noticed that about 42% of RC has a co-occurrence of *KRAS* and *TP53* mutation, while 32% of RC has a co-occurrence of *KRAS* and *APC* mutations [123]. The association of different types of mutations in the driver gene could be the reason for complexity or a better prognosis that needs better evaluation. Understanding cross-talk between different types of driver mutations could fetch valuable information in implementing treatment management. 

## 4. Prognostic Contribution and Therapeutic Outcome Associated with KRAS Mutation

**Figure 9:**
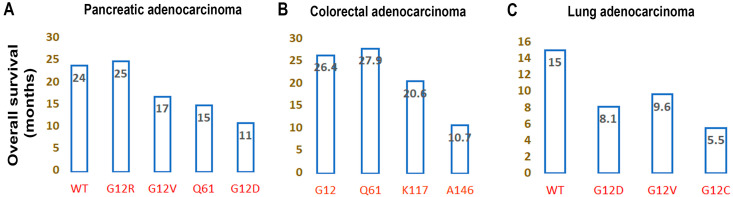
The median overall survival (OS) of KRAS mutant-harboring patients in different types of cancer. The column diagram represents the median OS of KRAS-mutated variants of pancreatic adenocarcinoma, where G12D shows worse survival compared to wild-type (WT) and G12R-mutated variants of KRAS [129] (**A**). In the case of colorectal adenocarcinoma-mutated KRAS variant at A146 shows poor survival compared to the mutated variants at G12 and Q61 [145] (**B**). In contrast, in Lung adenocarcinoma, patients harboring G12C mutation show worse survival compared to WT-KRAS (**C**) [159].

KRAS is one of the frequent driver mutations in several human cancers. Due to the complexities associated with KRAS-targeting drugs, different therapeutic regimens have been studied in clinical trials to address the efficacy of their therapeutic response. KRAS acts as both a prognostic and predictive marker in various types of cancer [124,125]. In human cancer, PAAD patients are among the highest populations affected by KRAS, followed by CRC and LUAD. The report suggests that patients who have KRAS mutation are associated with poorer prognoses in pancreatic cancer [126]. The meta-analysis study on pancreatic cancer reported that both the Caucasian and Asian populations had KRAS mutation with poor overall survival (OS). This study suggested that KRAS could help stratify high-risk patients for better therapeutic management. Bournet et al., in the study, observed no difference in OS among the mutant (8 months; 95% confidence interval (95% CI: 8.7–12.3) and WT KRAS (9 months; 95% CI: 8.7–12.8; hazard ratio HR: 1.03; *p* = 0.82) in advanced pancreatic cancer patients [127]. However, KRAS^G12D^ had a significantly shorter OS of 6 months compared to WT (9 months), KRAS^G12V^ (9 months), and KRAS^G12R^ (14 months). Similarly, Diehl and colleagues reported that PDAC patients with G12R KRAS variants had a longer OS compared to other KRAS mutant variants (20.4 versus 14.5 months, *p* = 0.0215) [128]. They also observed shorter OS among patients with concurrent G12R KRAS and PI3K mutation compared to the wild-type PI3K PDAC patients (19.4 versus 24.2 months, *p* = 0.057). Recently, the prognostic implication of KRAS mutation statuses and subtypes relative to KRAS^WT^ to determine OS of PDAC patients (*p* < 0.001) was mentioned by Yousef and his colleagues [129]. Considering all the stages, the median OS of WT KRAS patients had a longer survival duration, compared to the study by Bournet and colleagues. Similar to the Bournet’s group study observations, they also viewed KRAS^G12R^ variants had a longer median OS than KRAS^G12D^- and KRAS^Q61^-mutated tumors (G12R versus G12D: :34 versus 20 months; G12R versus Q61: : 34 vs. 22 months) [129]. In Stage IV, only PDAC patients had comparatively less OS (WT versus G12D: 24 versus 11 months) (Figure 9A) [129]. Quite contradictorily, a retrospective analysis of PAAD patients revealed that patients that have both KRAS^G12D^ mutation and TP53 mutation conferred better OS than other point mutations of KRAS (G12V/R/others), along with the TP53 mutation [130]. In patients with *KRAS*, *TP53*, *SMAD4*, and *CDKN2A* mutations, the coexistence of all four mutations had poor overall survival [130]. In the Phase III trial AIO-PK0104, comparing gemcitabine/erlotinib, followed by capecitabine and capecitabine/erlotinib, as well as gemcitabine, observed no significant difference [131]. This suggests the role of KRAS as a prognostic biomarker more than the predictive one in the chemotherapy response of pancreatic cancer patients [131]. Recent observations lead to a promise regarding predictive responses to chemotherapy associated with KRAS mutations. In a small cohort of KRAS^mut^ variants of pancreatic cancer patients, a new treatment regime of combining gemcitabine and a second generation of MEK inhibitor (cobimetinib) observed improved progression-free survival (PFS) among KRAS^G12R^ variants compared to the group of KRAS G12D and G12V [132]. This needs further confirmation in recruiting more patients.

Similarly, in CRC, patients with KRAS mutation act as primary risk category patients and need better treatment management [124]. Comparing the mutations of Codon 12 and 13, Codon 12 mutated patients showed shorter OS than Codon 13 and WT KRAS in CRC [133]. Dinu et al. reported a significantly faster OS of CRC patients having Codon 13 KRAS mutation compared to the WT diagnosed at Stages I and II [134]. Like pancreatic cancer, patients with the KRAS^G12D^ mutant variant in CRC noticed poorer prognosis than other types of KRAS mutation [135]. In another study, Imamura and colleagues observed an inferior survival of KRAS^G12V^ variants compared to KRAS^WT^/BRAF^WT^ patients [136]. They did not find any influence of KRAS-codon 13 mutation in prognosis [136]. In the UK cancer network study, it was observed that G12C and G12V variants, both, had poorer median OS compared to the wild-type KRAS (24.9 versus 35.1 months) among metastatic and recurrent CRC patients [137]. In another study, a higher risk of recurrence was observed among G12V and G12C KRAS CRC patients compared to those with WT or G12A, G12D, and G12C tumors [138]. The multicentre CRC study reported no difference associated with KRAS mutation among histologic stages, tumor sites, gender, geographic location, or age [139]. However, the G12D mutation was more frequent in patients with an anastomotic recurrence (58.2%) than in patients with other types of recurrence [139]. In the small patient population group, it was noticed that the KRAS mutation of Codon 12 was more metastatic compared to the mutation of Codon 13 (69.2% versus 30.8%) [140]. The liver is the more sensitive metastatic organ than the lung in the case of CRC [140]. However, the survival rate of liver metastasis was higher (46.7%) than lung metastasis (30.9%) [140]. Similar to the observations made by Jones and colleagues, Damit et al. observed the median survival of 25 months for KRAS^mut^ variants, compared to 35 months of KRAS wild-type metastatic CRC (mCRC) [140]. They further noticed that mCRC patients with KRAS mutations G12D, G12S, and G13D had a median survival of 23, 25, and 29 months, respectively [140]. The G12D mutation was most frequently associated with mCRC [140]. Santini et al. showed that KRAS^G12V^ was associated with hepatic metastasis [141]. Though there are different studies on KRAS mutation to predict the recurrence and survival of CRC, prognostic implications of mutation of the *KRAS* gene were not well defined. In colorectal liver metastasis (CRLM), Margonis et al. claimed that KRAS Mutations G12V and G12S had worse OS compared to KRAS^WT^ [142]. Moreover, the patients with recurrence after curative surgical liver resection, G12V, G12C, and G12S variants were associated with an increased death rate compared to the WT KRAS [142]. Likewise, Serebriskii et al., in their study, observed that the overall frequency of KRAS alterations increased in Microsatellite stable (MSS)/tumor mutation burden low (MT-L) patients with age, whereas microsatellite instability-high/tumor burden high (MT-H) patients had reduced alterations of KRAS with age [143]. According to the CRC 2023 statistics of the USA, the incidence rate of CRC increased by about 80–100% for the <50 age group, whereas this is about 20–30% for the age group 55–59 years and older [144]. Serebriskii and colleagues further showed that G12 mutations were prevalent among young patients (<40 years), whereas A146, K117, and Q61 were mainly commonplace in old age patients (>40 years) [143]. Within G12 variants, the frequency of G12V fractions increases with age, whereas G12A and G12C substitution frequency decreases [143]. The study group did not observe any changes in G13 mutation with the patient’s age, while the substitution of Q61 doubled with age (5.2% for >40 years compared to 2.5% for <40 years) [143]. Though the frequency of A146 mutation in mCRC with liver metastasis is less than the G12 mutation (7.7% versus 71.8%), A146 variants observed a higher level of mutant copies per mL of plasma (MTc/mL) than G12 variants (value of median MTc/mL of 35,338 vs. 700) identified through droplet digital polymerase chain reaction of liquid biopsy samples [145]. Moreover, mutant allele frequency (MAF) and total tumor volume (TTV) were 2.5-fold and 6-fold higher, respectively, in the case of A146 than in G12 variants [145]. The increased plasma-circulating tumor DNA (ctDNA) of KRAS-A146 mutation noticed high plasma ctDNA levels of TP53, telomerase reverse transcriptase (TERT), and PIK3CA, which indicates the association of high tumor burden with A146 mutation [145]. Those KRAS^A146^ mutation-harboring patients had significantly shorter survival than KRAS^G12^ mutation (median value of 10 versus 26.4 months) [145] (Figure 9B). For the lung metastasis of CRC patients, Codon 13 plays a significant role in poor prognosis [146]. Luo et al., in their study, reported that the bi-mutations of KRAS and PIK3CA among CRC patients were associated with poor overall survival [147]. In the same study, the multivariate analysis observed a high risk of death of CRC KRAS exon 3 or 4 mutated patients compared to exon 2 KRAS mutations with PIK3CA bi-mutation (univariate Hazard Ratio (HR) = 8.05; 95% confidence interval (CI): 1.926–33.64, *p* = 0.004; multivariate HR = 10.505; 95% CI: 2.304–47.905, *p* = 0.002) [147]. The concurrent presence of PIK3CA mutation and KRAS mutation also act as predictive biomarkers in therapeutic management [93]. Not only as a prognostic biomarker, but KRAS mutation also acts as a robust predictive biomarker of anti-EGFR therapy [148]. The anti-EGFR treatment (cetuximab and panitumumab) is mainly effective with WT RAS [149], whereas tumors harboring *KRAS* mutations in Exon 2 (Codons 12 and 13), Exon 3 (Codons 59 and 61), and Exon 4 (Codons 117 and 146) do not gain benefit from anti-EGFR therapy [150,151,152]. Though contradictory, not all KRAS mutants confer resistance to anti-EGFR therapy. In the retrospective study, comparing different types of KRAS mutations, patients bearing KRAS^G13D^ variants had extended OS (G13D versus other KRAS mutations: median 7.6 versus 5.7 months, HR, 0.50), as well as progression-free survival (PFS) [47]. In contrast, the combination treatment of cetuximab and chemotherapy showed longer OS and PFS of KRAS^G13D^ mutant variants compared to other KRAS mutants (OS: median 10.6 versus 7.4 months, HR 0.46; PFS: median 4.1 versus 2.8 months, HR, 0.49) [47]. The therapeutic benefit of harboring the G13D mutation could be due to the weak transforming potential, which was evidenced through in vitro experimental conditions [47]. Chemotherapy is the standard treatment approach for CRC, where a combination of 5-fluorouracil, leucovorin, and oxaliplatin (FOLFOX) is used to treat the patient. KRAS G12D mutation could act as a predictive biomarker for inferior FOLFOX response and a high risk of recurrence [153]. In the case of locally advanced rectal adenocarcinoma, KRAS status determines the therapeutic response of neoadjuvant chemoradiation therapy [154]. The pathologic complete response (pCR) rate was significantly lower in KRAS^mut^ patients (13%) compared to KRAS^WT^ patients [154].

In the case of lung cancer, the *KRAS* mutation is associated with the histology of cancer (adenocarcinoma versus squamous cell carcinoma: 37.2% versus 4.4%), smoking history of the patients (smokers versus non-smokers: 30% versus 11%), gender (female versus males: 31.35% versus 23.7%), and the ethnicity (Caucasian versus Asian: 26% versus 11%) [155,156]. The mutation of *EGFR* is the major factor associated with non-small cell lung carcinoma (NSCLC), in addition to a frequent mutation of *KRAS* and fusion of anaplastic lymphoma kinase (*ALK*) [157]. However, the mutation of *KRAS* is mutually exclusive with *EGFR* mutation and *ALK* fusion [158]. Like pancreatic and colorectal carcinoma, the *KRAS* mutation acts as a prognostic and predictive biomarker in NSCLC and metastatic NSCLC [125,159]. Scheffler and his colleagues observed the median OS of 52.6, 59.3, and 12 months of 609 recruited patients harboring *KRAS* mutation with Stages II, III, and IV, respectively [92], while Stage I patients did not reach the OS stage within the study period. However, in the whole cohort, different KRAS mutant variants of Stage IV did not show any variability in their median OS [92]. Sun et al. in their study observed that among 484 advanced NSCLC patients, 38% had *EGFR* mutation, whereas 8% had *KRAS* and only two patients had both *KRAS* and *EGFR* mutations (co-mutation of KRAS/EGFR were G12V/deletion in exon nine and G12D/L858R) [159]. Further evaluating the survival analysis among these two types of mutation, patients were stratified as *EGFR^WT^KRAS^WT^*, *EGFR^mut^KRAS^WT^*, and *EGFR^WT^KRAS^mut^*. The median OS was 15, 38, and 7.7 months in the groups of WT for both the factors, *EGFR* mutation, and *KRAS* mutation, respectively [159]. Among different types of KRAS mutant variants (G12D, G12V, G12C, and others) there were no significant differences in the OS of the patients (Figure 9C). However, this study predicted the effect of tyrosine kinase inhibitor (TKI) therapy within the study group, where the EGFR responsiveness of TKI therapy was better among *KRAS^WT^* compared to *KRAS^mut^* (*KRAS^mut^* vs. *KRAS^WT^*: 14% vs. 56%, *p* = 0.002) [159]. The study group also observed the effectiveness of pemetrexed-, gemcitabine-, and taxane-based first-, second-, and third-line chemotherapy, respectively. The pemetrexed-based treatment regimen observed a lower response rate of chemotherapy compared between WT and mutated variants of KRAS patients (*KRAS^WT^* versus *KRAS^mut^*: 28% versus 14%), besides their shorter progression-free survival (PFS) (*KRAS^WT^* versus *KRAS^mut^*: 3.9 versus 2.1 months) [159]. The gemcitabine-based treatment approach also noticed a similar response of lower response rates among mutated variants of KRAS patients compared to WT KRAS patients (*KRAS^WT^* versus *KRAS^mut^*: 36% versus 18%), in addition to PFS (*KRAS^WT^* versus *KRAS^mut^*: 4.2 versus 2.4 months). The taxane-based regimen showed no difference in the therapeutic response among the KRAS mutant patients [159]. In the single institution-based study, KRAS^G12C^ patients at diagnosis showed poorer performance than other mutated variants of KRAS [160]. Further, KRAS^G12C^ patients showed a lower OS of less than 12 months [160]. Ihle et al., in their study, noticed that patients with refractory NSCLC that have G12C and G12V mutant variants had poor PFS (median survival = 1.84 months, *p* = 0.046) compared to the mutant variants G12A and G12D (median survival = 3.35 months) and patients with WT KRAS (median survival = 1.95 months) [68]. In the TRAILOR trial, the highest incidence of KRAS mutation reported among NSCLC patients was G12C, followed by G12V, G12D, and G12A among NSCLC patients [34]. This trial reported that patients with KRAS mutations had lower survival compared to the patients with WT KRAS (unadjusted Hazard Ratio [HR] = 1.41 95%CI: 1.03–1.94, *p* = 0.032; adjusted HR = 1.39 95% CI: 1.00–1.94, *p* = 0.050). Moreover, the study claimed that the presence of KRAS mutations could be the reason for the negative impact on OS with first-line platinum-based chemotherapy [34]. This study did not find any survival benefit between different KRAS mutations. In another study, the combination of platinum and taxane showed an improved overall response rate (ORR) (50%) among KRAS^G12V^ variants, compared to platinum + gemcitabine (25%) and platinum + premetrexed (21%) [161]. The addition of bevacizumab with platinum + taxane had the highest ORR (62%). No significant improvement of ORR was noticed in other G12-mutated patients in this study. Though the use of taxane showed significantly improved ORR (*p* = 0.01) for G12V mutant variants, progression-free survival (PFS) and OS did not show any beneficial effect [161]. In a different study, a comparatively significant number of retrospective patients of Stage IV NSCLC was evaluated for the effectiveness of platinum-based first line of chemotherapy with treatment arms (pemetrexed, vinorelbine, gemcitabine, taxane, or bevacizumab) for OS and time to progression (TTP) [162]. Taxane showed the best response in the entire cohort (OR: 2.52 (95% CI: 1.82–3.48), *p* < 0.001), more specifically, G12V patients (OR: 2.15 (95% CI: 1.05–4.41), *p* = 0.036) [162]. An improved level of TTP was also noticed with the use of taxane (HR: 0.31 (95% CI: 0.26–0.38), *p* < 0.001), where G12V-mutated variants noticed significantly improved level of TTP (HR: 0.47 (95% CI: 0.22–1.01), *p* = 0.054). Treatment with pemetrexed noticed worse levels of TTP, including the G12V mutated variants (HR: 0.55 (95% CI: 0.30–0.99), *p* = 0.049) [162]. OS remained indifferent among KRAS mutant variants with the treatment regimen following different chemotherapeutic drugs. In the BATTLE trial, for an 8-week disease control rate (DCR), treatment with sorafenib observed impressive benefits compared to treatment with erlotinib or bexarotene plus erlotinib patient group among KRAS mutant patients compared to the WT KRAS variant patient population [163]. Smoking habits among the NSCLC patients were also significantly associated with the KRAS mutation (*p* = 0.001) [164]. Patients that have smoking habits frequently observe the KRAS mutation of G12C/G12A/G12V/G13C, whereas non-smokers are G12D/G12S/G13D [155,165].

## 5. Therapeutic Strategy against KRAS Mutations

**Figure 10:**
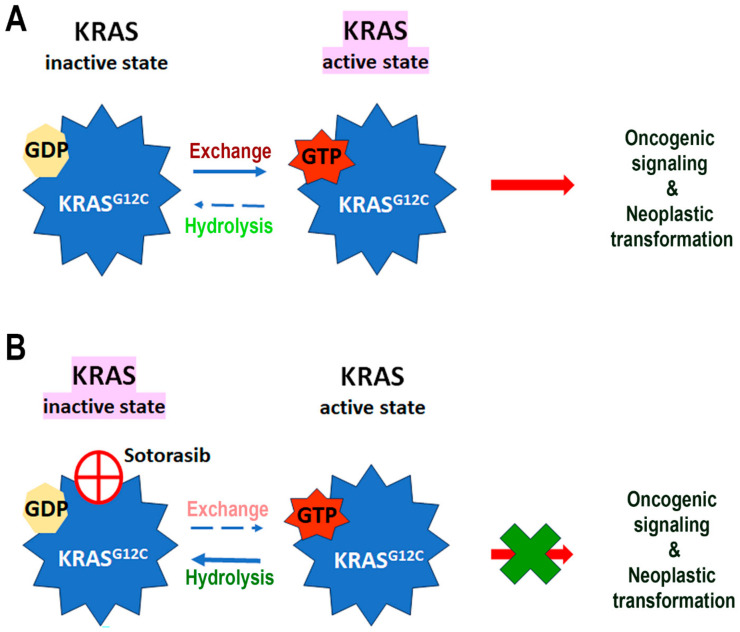
Mechanism of inhibition of mutated-KRAS using Sotorasib inhibitor. (**A**) A mutated form of KRAS^G12C^-GDP inactive state activated by guanine nucleotide exchange factor (GEF) to KRAS^G12C^-GTP active state. KRAS^G12C^-GTP is the cell’s major form due to disrupted GTPase-activating protein (GAP), which converts to KRAS^G12C^-GDP state. The active form of KRAS^G12C^-GTP regulates oncogenic signaling leading to neoplastic transformation. (**B**) Sotorasib binds explicitly to the KRAS^G12C^-GDP inactive state of the protein and restricts the conversion of the KRAS^G12C^-GTP state leading to the trapping of KRAS^G12C^-GDP. With the restriction of the KRAS-active state, there is inhibition of oncogenic signaling and neoplastic transformation.

Due to the complexities of the RAS pathway, it is puzzling to target specific upstream druggable factors, the region of KRAS protein, or the interaction complex, to ensure the best therapeutic outcome. Lacking targetable structural pocket in the KRAS protein and having a high affinity to GTP, earlier, KRAS was considered an undruggable oncoprotein to be targeted. After an extensive study on KRAS, only recently, in 2021, two promising inhibitors against mutated KRAS (KRAS^G12C^) were clinically approved by the Food and Drug Administration (FDA) of the USA and the European Medicine Agency to treat advanced NSCLC patients [124,125]. Mutation-specific druggable targets of KRAS observed significant impact after its potential beneficial outcome using sotorasib and adagrasib for NSCLC KRAS^G12C^ mutant patients [9,166,167]. Targeting the structural plasticity of the switch region provided success in managing the historical undruggable characteristics of KRAS with the covalent G12C inhibitors [22]. In this pioneering work of K Shokat’s laboratory in 2013, the study group screened about 480 cysteine-reactive small molecules, targeting the nucleophile thiol group of Cys, which bound explicitly with the covalent compound KRAS^G12C^-GDP [168]. X-ray crystallography revealed that KRAS^G12C^ binding with the inhibitors disrupts the interaction between Switch I and II, besides favoring the nucleotide preference of GDP over GTP with the mutated protein [168]. Moreover, strike compounds enable the expansion of the Switch II pocket and the exposure of mutant Cys by displacing glutamine 61 [168]. The success of this discovery was based on its unique strategy, where the nucleophilic property of Cys thiol was targeted by the Cys-reactive small molecules (CrSM). This property of KRAS^G12C^ provided an added advantage over the KRAS^WT^ protein. The CrSM binds at the allosteric site of the protein, which was evidenced by the simultaneous binding of 1mM of GDP in the experimental condition [168]. This suggests that KRAS has a non-overlapping site of GDP binding and binding of CrSM. The covalent binding of CrSM at Switch Pocket II leads to conformational changes and favors the binding of GDP over GTP, which reduces the active state of KRAS and checks further downstream pathways. Figure 10 shows the binding of sotorasib with the KRAS^G12C^-GDP state with the allosteric site of the protein and preventing the conversion of the KRAS^G12C^-GTP active state. The strategy developed to inhibit G12C with sotorasib was initially identified while studying the nuclear magnetic resonance using a small molecule inhibition of the Switch II pocket, hence leading to the identification of a sugar derivative ligand, SCH-54392 [169,170]. Despite its limited affinity, studies guided the implementation of an alternative procedure of targeting the allosteric site instead of directly dealing with GDP or GTP with KRAS. Eventually, three potent G12C inhibitors were explored for the clinical trial, namely Sotorasib (AMG510) from Amgen [171,172], Adagrasib (MRTX-849) from Mirati, and JNJ-74699517 [173,174,175]. The success of G12C was equally inspired to address other mutants of KRAS by using the covalent ligands for their therapeutic efficacy. Recent development of covalent ligands to target G12S and G12R needs to be noted. Characterization of small molecule compound for G12S ligand, an electrophilic group, β-lactone reacts explicitly with a serine residue, which could attach with the tetrahydropyridopyrimidine moiety of G12C ligand Adagarisib [176]. These covalent ligands of G12S, G12Si-1/5, could inhibit the loading of GTP with KRAS besides restricting the phosphorylation of ERK. Similarly, a compound containing α, β-nikethamide could be covalently bound with the KRAS^G12R^–GDP state by interacting with the nitrogen side chain of Arginine 12. Despite the attachment of the small molecule compound, the predominant form of KRAS^G12R^ remained in an active GTP state; this compound was not as effective as the G12S ligand, which needs further study for the identification of an effective G12R inhibitor [177].

There is an urgent need for beneficial therapeutic effects while targeting KRAS^G12D^; this G12D mutation is one of the most prevalent mutated forms of KRAS in both pancreatic and colorectal cancer (Table 1). The pertinent question of targeting the G12D, and other mutations arises as to whether a similar strategy could be applied to G12C. Unlike G12C, other mutations (G12D, G12V, G12R, G12S, and Q61H) lack active residue, like cysteine. Therefore, this requires a novel approach to noncovalently block the amino acid at Codon 12. The second challenge associated with the G12X mutation is that it lacks the intrinsic hydrolysis activity of G12C. Therefore, G12X is used to remain in a GTP-bound state [178]. The discovery of BI-2852 effectively targets the GTP-bound “ON” state and GDP-bound “OFF” state of KRAS-G12D [179]. This compound binds to a pocket between Switch I and Switch II, blocking effectively all GEFs, GAP, and effector interaction leading to the dampening of downstream signals and restricting proliferation. BI-2852 is a useful chemical probe for studying RAS biology in an in vitro setting. In this effort to target G12D, other efforts are continuing for the effective management of KRAS G12D, where synthetic chemicals were selected and screened for targeting the 12-aspartate moiety. Zeng and colleagues screened such compounds that have considerable affinities with the Switch Pocket II as well as targeting selectively the 12-aspartate moiety [180]. The key idea of targeting 12-aspartate is to introduce a salt bridge between the SII-pocket and aspartate to compensate lack of acrylamide-cysteine 12 covalent bond of KRAS^G12C^ [180].

The structure-based drug design discovered MRTX1133, a potent, selective KRAS^G12D^ noncovalent compound inhibiting downstream signaling events in vitro and in the mouse model system [181]. MRTX1133 showed higher affinity towards the KRAS^G12D^-GDP state of the protein with a dissociation constant (K_D_) and drug inhibitory (IC_50_) values of about 0.2 pM and less than 2 nM, respectively [182]. MRTX1133 can target both the active and inactive states of KRAS. This compound is selective for the mutant KRAS^G12D^ compared to the KRAS^WT^, which inhibits phosphorylation of ERK1/2, besides tumor regression in the mutant cell-line-derived and patient-derived xenograft model of PDAC [182]. However, Feng et al. recently noticed that KRAS^G12D^-mutated CRC cells treated with MRTX1133 observed downregulated expression of a negative regulator of EGFR, which in turn caused EGFR-feedback activation [183]. This feedback activation of EGFR by MRTX1133 reduced the molecule’s efficacy to target G12D, which needs further study. In this screening process of the non-covalent inhibitor to target G12D, G12V, G13D, and Q61H, a new component was identified as ACA-14 (2-hydroxy-5{[(2-phenyl-cyclopropyl)carbonyl]amino} benzoic acid) by Pagba et al., which showed promise in the development of broad acting inhibitors targeting different types of KRAS mutations [184]. The study group noticed that ACA-14 was able to bind with KRAS^WT^ and mutated KRAS, including KRAS^G12D^, in a nucleotide-independent manner and inhibited both the intrinsic and GEF-mediated GTP/GDP exchange rates, as well as directly disrupted the effector binding. Furthermore, ACA-14 inhibited KRAS^G12D^ downstream MAPK signaling in the baby hamster kidney (BHK) cells and reduced the growth of the tumor in the MIAPaCa-2 xenograft mouse model driven by KRAS^G12C^ mutation [184]. This inhibitor specifically inhibited the KRAS by targeting the pocket formed by three N-terminal β-strands and the switch region of KRAS, or between the Helices 2 and 3, but not NRAS or the HRAS [184]. Tested for different types of KRAS mutated cell lines of pancreatic cancer [MIAPaCa-2 (G12C), MOH (G12R), PANC-1 (G12D)], and colon cancer [SW116 (G12A), SW948 (Q61L)], which observed significant inhibition of proliferation of cancer cells [184]. To address the issue of effectiveness in targeting the inactive state of non-G12C KRAS mutations, various small molecule inhibitors were screened by Kim et al. [20]. Using the structure-based designing approach, the study group discovered a pan-KRAS inhibitor from the G12C selective inhibitor BI-0474 [20]. This pan-KRAS inhibitor (pan-KRASi) (BI-2865) could block the activation of different KRAS mutants, namely, G12A/C/D/F/V/S, G13C/D, V141, L19F, Q22K, D33E, Q61H, K117N, and A146V/T [20]. Using isothermal titration calorimetry assay, it was observed that the inhibitor could bind with the WT, G12C, G12D, G12V, and G13D KRAS–GDP state with high affinity (K_d_ = 10–40 nM), which was significantly lower than the KRAS variants loaded with the GTP analog GCP [20]. A significantly higher concentration of inhibitor was used to prevent the effector binding of active KRAS than its inactive state (IC_50_ value of 5.5 μM versus 5 nM). This pan-KRASi showed an equal potency of deactivation of KRAS like the sotorasib, the KRAS^G12C^ inhibitor [20]. The level of activated KRAS-GTP was diminished, while treatment with 10nM of pan-KRASi for 2 h of incubation in the in vitro model of different pancreatic, lung, and colorectal cell lines was also diminished. The in vitro analysis of targeting the signaling pathway in various types of KRAS mutated cell lines observed the inhibition of the phosphorylation of ERK (IC_50_ value of about 150 nM for pERK) and RSK (IC_50_ value of about 70 nM for pRSK) [20]. A similar observation was noticed in HEK293 while using the pan-KRASi. Kim et al. used different types of KRAS-mutated cell lines (Table 3) to understand the effectiveness of pan-KRASi in the preclinical model [20]. In the lung adenocarcinoma PC9 cell line, inhibitors could influence upstream activation signaling, evidenced by diminished KRAS–GTP levels [20]. This indicates that pan-KRASi can target the downstream and upstream events of the KRAS-signaling mechanism.

There are also indirect strategies for halting KRAS from the cell’s neoplastic transformation. To restrict KRAS-mediated neoplastic transformation in the cell, various well-known indirect strategies are being implemented, significantly (i) inhibiting KRAS from posttranslational modification, (ii) displacing KRAS protein from the plasma membrane, (iii) deterring ligands from interacting with RTKs, (iv) restraining the RTK interactive partners towards downstream activation, and (v) inhibiting downstream effectors of KRAS [185]. In a posttranslational modification, the series of prenylation at the carboxy-terminal HVR leads to KRAS localization and attachment with the membrane [186]. Prenylation is a cascade of enzymatic steps; impairment of any stage of this process will hamper the localization of KRAS to the plasma membrane. At the first step of this process, farnesyl (C15) isoprenoid moiety is attached to the Cys in the CAAX motif of KRAS by farnesyl transferase (Ftase) [187]. Studies noticed an alternative attachment of C20 geranyl isoprenoid moiety by geranyl geranyl transferase (GGTase), which could be activated if farnesylation is inhibited [185]. After adding C15 farnesyl isoprenoid moiety, terminal AAX amino acids are cleaved by the protease RAS-converting enzyme 1 (RCE-1) [186,187]. Then isoprenyl cysteine carboxymethyl transferase (ICMT-1) catalyzed the methylation of the carboxyl group of Cys [186,187]. That prenylation process makes KRAS more hydrophobic and reduces its solubility, in turn facilitating the localization and attachment at the membrane with the help of phosphodiesterase-δ (PDE-δ) [185]. This series of enzymatic steps has attracted researchers to design a therapeutic strategy to inhibit oncogenic KRAS towards reaching the membrane. The Ftase inhibitors (FTI-277, B956, Lonafarnib, Tipifarnib) were effective in preclinical colon, pancreatic, and lung carcinoma studies. However, the clinical study did not show promise [185,188]. The strategy of blocking farnesylation could not be effective because there is an alternative pathway of geranylation, which eventually helps in the downstream effect of posttranslational modification leading to KRAS localization towards the membrane. Cysmethynil, an ICMT inhibitor in mouse embryonic fibroblast, impairs membrane localization and epidermal growth factor-mediated signaling [189]. However, further study is essential to improve the effectiveness and safety of ICMT inhibitors [190]. Regarding the displacement of RAS from the membrane, salirasib and fendiline showed promising outcomes for further clinical study [191]. In the mouse xenograft model of pancreatic cancer, salirasib and gemcitabine inhibited tumor growth by displacing KRAS from the membrane and influenced the reduction of phosphorylation of MAPK and AKT [192,193]. The in vitro study of salirasib and celecoxib, a cyclooxygenase inhibitor, strongly inhibited NFκB activity, reducing pAKT and Bcl-2 in pancreatic cell lines [194]. Dose-limiting toxicity and safety trials among Japanese patients of relapsed/refractory solid tumors including pancreas, colorectal, and biliary tract tumors noticed a safe and well-tolerated oral use of salirasib, which was referred for Phase II study [195]. Fendiline, a calcium channel blocker, inhibited the explicit localization of KRAS at the membrane, which impacts the activation of AKT and ERK, as well as the reduction of c-myc and CD44 expression in the pancreatic cancer cell line (PANC1, MIAPACA2, CD18) [196,197]. Both PANC1 and MIAPACA2 display mesenchymal characteristics, whereas CD18 is epithelial [197]. Newly discovered fendiline analogs to optimize their drug properties indicated their potential in anticancer therapeutics [198]. In the last step of KRAS landing on the plasma membrane, perturbing KRAS/PDE-δ could be a tractable therapeutic strategy in restricting the tumor neoplastic growth and downstream effector pathways. Deltarasin, a small molecule inhibitor, blocks the interaction between the KRAS and PDE-δ at the hydrophobic pocket of their interactions, which eventually restricts KRAS from being delivered at the PM [199]. Though deltasarin inhibits the cell’s autophagy, besides increasing reactive oxygen species, the inhibition of PDE-δ for the KRAS therapeutic strategy needs further validation to reach the clinic [191]. While deterring the ligand from interaction with RTK, cetuximab, and panitumumab, these two monoclonal antibodies (mAbs) bind with the EGFR at the extracellular Region III and prevent binding of the ligand with the RTK and lock the receptor in its monomeric autoinhibitory conformation [149]. Though their mode of function is different, these two drugs are primarily used to treat RAS wild-type metastatic colorectal cancer either as monotherapy or in combination with chemotherapy [149]. There is also intracellular targeting of RTK, where erlotinib, afatinib, and lapatinib could influence KRAS-mediated pathways by influencing the intracellular tyrosine kinase domain [200]. In the strategy of therapeutic development against wild-type KRAS, the downstream factors of RTK, namely the GEF, adaptor protein, and SOS1-activating non-receptor protein tyrosine phosphatase SHP2, are also included in the therapeutic strategy [201,202]. In this indirect strategy of KRAS suppression, several inhibitors were used to target SOS1 and SHP2 [182,196,197]. However, their efficacy is challenging in targeting KRAS-mutated tumors. Besides targeting the upstream factors, downstream effector molecules are also targeted in suppressing KRAS-mediated neoplastic transformation [188,201,202]. The well-known RAF (LXH-254, Lifirafenib), and PI3K (Alpelisib, Copanlisib, Duvelisib, Idelalisib) inhibitors are under clinical trials to treat different types of cancer [188]. LXH-254 demonstrated anti-cancer activity in tumors with BRAF/RAS co-mutations. However, there was moderate activity against KRAS mutations [203]. In the preclinical study, lifirafenib showed effectiveness in antitumor activity along with the MEK inhibitors [204]. It has been observed that RAF inhibitors are not effective with KRAS-mutated patients. Alpelisib is an FDA-approved drug for treating solid tumors, while the other above-mentioned PI3K inhibitors are used against relapsed or refractory follicular lymphoma with RAS mutations [188]. Similar to RAF inhibitors, PI3K also has limited benefits to target KRAS mutated tumors. There are also MEK, ERK, AKT, and MTOR inhibitors [188]. However, it needs more study for their effectiveness as a clinical agent. This suggests that direct and indirect strategies for treatment management to target KRAS mutations are still unachievable. However, the progress to target G12C using sorafenib and preclinical studies on pan-KRASi could provide beneficial effects in future therapeutic management.

## 6. Conclusions

The untargeted or uncontrollable characteristics of KRAS remained in its inner complexity, which provides the benefit of advantage in regulating diversified effector pathways and downstream targets in the neoplastic transformation. Moreover, substituting amino acids at the hotspot region converts KRAS to loss of the inner GTPase activity or impaired GTPase property by the GAP enzyme leading to their constitutive activation and modulating downstream MAPK, PI3K/AKT, and RAL pathway. Among different variants of KRAS mutation, G12D and G12V are the predominant mutations associated with PAAD and CRAD, whereas G12C is predominant in LUAD. Specifically, in rectal adenocarcinoma, a higher prevalence of G12V was noticed compared to colon adenocarcinoma. Those mutations also play a significant role in prognosis and in predicting the therapeutic response. Both G12D and G12V lead to a worse prognosis and less response towards chemotherapy, while G13D has a better prognosis and response towards therapy. In CRC, shorter survival associated with A146 compared to the G12 mutation needs further study. The cell culture and animal model study of different types of KRAS mutations noticed different signaling pathways, which could be the probable reason for the different intensities of cancer pathology associated with KRAS mutations. The use of different cell lines harboring the specific KRAS mutation could help in a better understanding of molecular factors associated with neoplastic transformation. Not only specific KRAS mutations could influence the pathological nature of cancer or its therapeutic response, but also the co-occurrence and correlation of other driver mutations with KRAS could significantly influence OS, PFS, or disease recurrence and therapeutic management. Though G12D and G12C are predominant among pancreatic, colorectal, and lung adenocarcinoma, G12V is the prevalent mutation of KRAS, which is associated with the co-occurrence of MTOR, PIK3CA, TP53, and APC mutations. The co-occurrence of KRAS and other driver mutations could be detrimental or beneficial, which needs a better screening strategy to understand the pathology of cancer. This could have immense significance as a prognostic and predictive marker in treatment management.

Direct targeting of the KRAS mutation is always advantageous over the indirect approach to target KRAS mutation-associated neoplastic growth and differentiation. In this direct strategy, the first success in targeting the KRAS mutation was achieved against KRAS^G12C^ in NSCLC, where sotorasib provides a better outcome. Targeting G12C was achieved by targeting the allosteric region at Switch Pocket II, where the thiol group-containing nucleophilic region was restrained with cysteine-reactive small inhibitors, instead of targeting the GDP-to-GTP transition. The sotorasib binds with the KRAS^G12C^-GDP state, which restricts the transition to the KRAS^G12C^-GTP state and further jams the activation of effector molecules for neoplastic transition. With the success in targeting the allosteric site of KRAS^G12C^, a similar strategy is being followed to target other KRAS mutations, specifically the G12D, which is notorious among the KRAS mutations. Targeting aspartic acid needs a modified approach or the specific small molecule inhibitors are yet to be identified. Another approach to check KRAS^mut^ activation is screening for pan-KRAS inhibitors. However, it is also at the level of the preclinical stage. A multiprong approach is necessary to control the neoplastic transformation of the cell regulated by KRAS mutations. Different KRAS mutations modulate different types of downstream effector pathways. Integrating specific signaling pathways of mutated-KRAS and crosstalk between driver mutations or effector factors will help in treatment management and implementation of the best therapeutic strategy.

## Figures and Tables

**Table 1:** List of KRAS mutations in different types of cancer followed by cBioPortal study.

Type of Cancer	KRAS Mutation (Relative Frequency in Percentages)
Pancreatic adenocarcinoma	G12D (41.80), G12V (27.04), G12R (21.31), G12A (0.8), G12C (0.8), G12S (0.8)
	G13C (0.8)
	Q61H (4.91), Q61R(1.63)
Colon adenocarcinoma	G12D (26.57), G12V (20.27), G12C (6.29), G12A (4.19), G12S (4.19), G12R (1.39),
	G13D (16.78), G13C (1.39)
	Q61H (1.39), Q61R (1.39), Q61K (1.39), Q61P (0.69), Q22K
	K117N (2.09)
	A146T (7.69), A155D (0.69), A146V (0.69)
	P34L (0.69), R68S (0.69), Y71C (0.69)
Rectal adenocarcinoma	G12V (30.0), G12D (20.0), G12C (10.0), G12A (6.0), G12S (2.0)
	G13D (18.0)
	A146T (6.0), A59T (2.0)
	Q61E (2.0), Q61H (2.0), Q61L (2.0)
Lung adenocarcinoma	G12C (40.90), G12V (23.39), G12D (11.69), G12A (9.94), G12S (2.90),
	G13C (4.09), G13D (1.75)
	Q61L (1.75), Q61H (0.58)
	L19F (1.75), A146P (0.58), D33E (0.58)
Lung squamous cell carcinoma	G12A (16.66), G12V (16.66)
	G13C (16.66)
	Q61H (16.66)
	E3K (16.66)
	V14I (16.66)
Uterine Endometroid carcinoma	G12D (34.04), G12V (20.21), G12A (8.51), G12C (6.38), G12S (2.12)
	G13D (10.63), G13C (3.19), G13V (1.06)
	Q61H (2.12), Q61L (1.06)
	A59G (1.06), A130V (1.06), A146T (1.06), A146V (1.06)
	I24N (1.06), K176Q (1.06), P110S (1.06), R164Q (1.06)
Stomach adenocarcinoma	G12D (14.28), G12S (14.28), G12C (4.76), G12V (4.76)
	G13D (42.85)
	A146T (4.76)
	Q61H (4.76), R135T (4.76), R151T (4.76)
Cutaneous Melanoma	G12D (12.5), G12R (12.5)
	G13D (25.0)
	K117N (12.5)
	M72K (12.5)
	S122F (12.5)
	L25R (12.5)
Acute Myeloid Leukemia	G12D (20.0), G12V (20.0)
	G13D (20.0)
	Q61H (10.0)
	A59E (10.0), A146T (10.0)
	I36M (10.0)
Hepatocellular carcinoma	G12D (50.0), G12C (25.0)
	G13D (25.0)
Bladder Urothelial	G12D (35.71), G12C (14.28), G12V (14.28), G12R (7.14),
	G13D (7.14)
	L19F (14.28)Q61H (7.14)
Cervical squamous cell carcinoma	G12V (28.57), G12C (14.28), G12D (14.28)
	G13D (28.57)
	I21R (14.28)
Endometrial adenocarcinoma	G12C (20.0), G12D (20.0)
	A146T (20.0)
Ovarian Serous Cystaadenocarcinoma	G12V (66.66), G12R (16.66)
	Q61L (16.66)
Uterine carcinosarcoma	G12V (42.85), G12D (28.57), G12A (14.28), G12C (14.28)
Breast Invasive Ductal Carcinoma	G12V (60.0), G12D (20.0)
	D92Y (20.0)
Breast Invasive Lobular Carcinoma	G12C (100.0)
Papillary Thyroid Cancer	G12V (25.0)
	Q61K (50.0), Q61R (25.0)
Prostate adenocarcinoma	G12D (50.0), G12R (50.0)
Cholangiocarcinoma	G12R (100.0)
Esophageal adenocarcinoma	G12D (100.0)
Astrocytoma	G12A (50.0)
	S17T (50.0)
Glioblastoma Multiforme	G12D (100.0)

Data collected from the TCGA pan Cancer Atlas in cBioPortal.

**Table 2:** Interaction and activation of downstream effectors associated with KRAS G12 mutant variants [8,49,50,69].

Characteristics	KRAS^G12D^	KRAS^G12C^	KRAS^G12R^	KRAS^G12V^	KRAS^G12A^
RAF1 Interaction	**++**	**+++**	**+**	**+**	**+++**
pERK1/2	**+++**	**++**	**+**	**+**	**+**
pMEK	**+++**	**+**	**–**	**–**	**–**
PI3K Interaction	**+**	**–**	**–**	**+**	**–**
pAKT	**+++**	**++**	**–**	**–**	**–**
pS6	**+++**	**+++**	**+**	**–**	**–**
RAL Interaction	**–**	**+**	**–**	**+**	**–**

In the table, pERK1/2: phosphorylated ERK1/2, pMEK: phosphorylated MEK, pAKT: phosphorylated AKT, pS6: phosphorylated S6, ‘+++’: very high, ‘++’: high, and ‘+’:present, ‘−’: not evaluated.

**Table 3:** List of available cell lines of KRAS mutations [20].

KRAS	Cell Lines
KRAS^WT^	NIH3T3, HEK293, MRC5, MRC9, BXPC3, A375, H1299, H520, H1975, PC9, H1650, HCC827, U87MG, U251MG, MEWO
KRAS^G12C^	H358, H2122, CALU1, MIAPACA2
KRAS^G12D^	ASPC1, PAC0403, HPAC, PANC1, LS513
KRAS^G12V^	SW620, SW480, H727, CAPAN1
KRAS^G12S^	A549
KRAS^G12R^	PSN1, PATC50, MOH
KRAS^G13D^	DLD1, LOVO, HCT116
KRAS^Q61X^	H460, CALU6
KRAS^K117N^	C125PM
KRAS^A146T^	WIL2NS, LS1034

## Supplementary Materials

The following supporting information can be downloaded at: https://www.mdpi.com/article/10.3390/cells13141221/s1, Figure S1: Mutant variants of KRAS with the coexisting mutations of MTOR in Pancreatic adenocarcinoma (PAAD), Colorectal adenocarcinoma (CRAD), and Lung adenocarcinoma (LUAD); Figure S2: Mutant variants of KRAS with the coexisting mutations of BRAF in Pancreatic adenocarcinoma (PAAD), Colorectal adenocarcinoma (CRAD), and Lung adenocarcinoma (LUAD); Figure S3: Diagrammatic presentation of crosstalk between Wnt and RAS signaling pathway.
