# Supplementary material for: KRAS Mutation Subtypes and Their Association with Other Driver Mutations in Oncogenic Pathways
Source: Cells. 2024 Jul 19;13(14):1221. doi: 10.3390/cells13141221 (PMC11274496; doi:10.3390/cells13141221)
Supplement: Supplementary file 1 [file cells-13-01221-s001.zip › cells-2838525-supplementary.pdf]

Supplementary Figure

KRAS mutation subtypes and their association with other driver mutations in oncogenic pathways

Koushik Mondal [1,2,*], Mahesh Kumar Posa [3], Revathi P. Shenoy [4] and Susanta Roychoudhury [1,5,*]

1 Division of Basic & Translational Research, Saroj Gupta Cancer Centre & Research Institute, MG Road, Kolkata-700063, West Bengal, India; mkoushik9@gmail.com

2 Department of Cancer Immunology, SwasthyaNiketan Integrated Healthcare & Research Foundation, Koramangala, Bengaluru-560034, Karnataka, India; mkoushik9@gmail.com

3 School of Pharmaceutical Sciences, Jaipur National University, Jagatpura, Jaipur, Rajasthan-302017, India; posa.mahesh@gmail.com

4 Department of Biochemistry, Kasturba Medical College, Manipal Academy of Higher Education, Manipal-576104, Karnataka, India; revathi.shenoy@manipal.edu

5 CSIR-Indian Institute of Chemical Biology, 4 Raja S.C.Mullick Road, Jadavpur, Kolkata-700032, West Bengal, India; susantarc@gmail.com

* Correspondence: KM (mkoushik9@gmail.com); SR (susantarc@gmail.com)

Supplementary Figures:

Supplementary Figure S1:

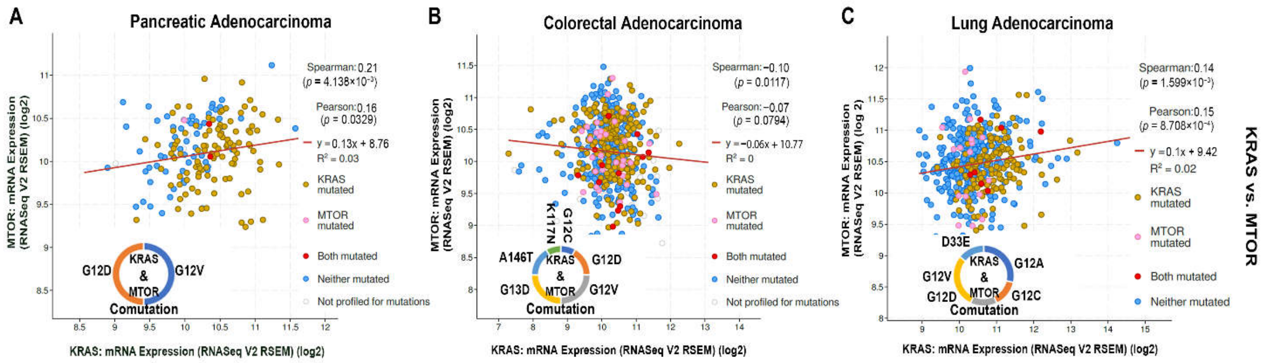

Figure S1: Mutant variants of KRAS with the coexisting mutations of MTOR in Pancreatic adenocarcinoma (PAAD), Colorectal adenocarcinoma (CRAD), and Lung adenocarcinoma (LUAD). In the Dot plots (A-C), mutated *KRAS* is represented as orange, mutated *MTOR* as pink, coexisting mutation of *KRAS* and *MTOR* as red, no mutation as blue, and not profiled for modification as white. The doughnut diagram (A-C) in each plot represents the KRAS mutation associated with the MTOR co-mutation. According to cBioPortal, G12D and G12V are the most prevalent mutations associated with MTOR co-mutation in PAAD (A). In the case of CRAD, G12C, G12D, G12V, G13D, A146T, and K117N KRAS mutations are associated with MTOR co-mutation. The prevalent KRAS mutations are G12V and G13D KRAS mutations (B). In contrast, G12A and G12V KRAS mutations are mainly associated with MTOR co-mutation in LUAD (C). Dot plots show no correlation between KRAS and MTOR in PAAD (A), CRAD (B), and LUAD (C). Data collected from cBioPortal.

Supplementary Figure

Supplementary Figure S2:

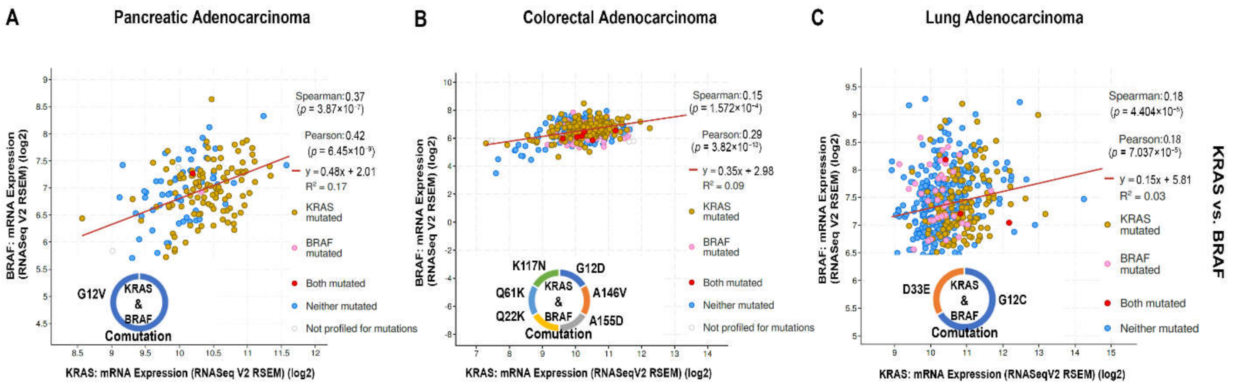

Figure S2: Mutant variants of KRAS with the coexisting mutations of BRAF in Pancreatic adenocarcinoma (PAAD), Colorectal adenocarcinoma (CRAD), and Lung adenocarcinoma (LUAD). In the Dot plots (A-C), mutated *KRAS* is represented as orange, mutated *MTOR* as pink, coexisting mutation of *KRAS* and *BRAF* as red, no mutation as blue, and not profiled for modification as white. The doughnut diagram (A-C) in each plot represents the KRAS mutation associated with the BRAF co-mutation. According to cBioPortal, co-muatation of KRAS and BRAF is mainly associated with CRAD. Though co-mutation exists in the CRAD, however, no significant correlation was observed between KRAS and BRAF in CRAD (B). Data collected from cBioPortal.

Supplementary Figure

Supplementary Figure S3:

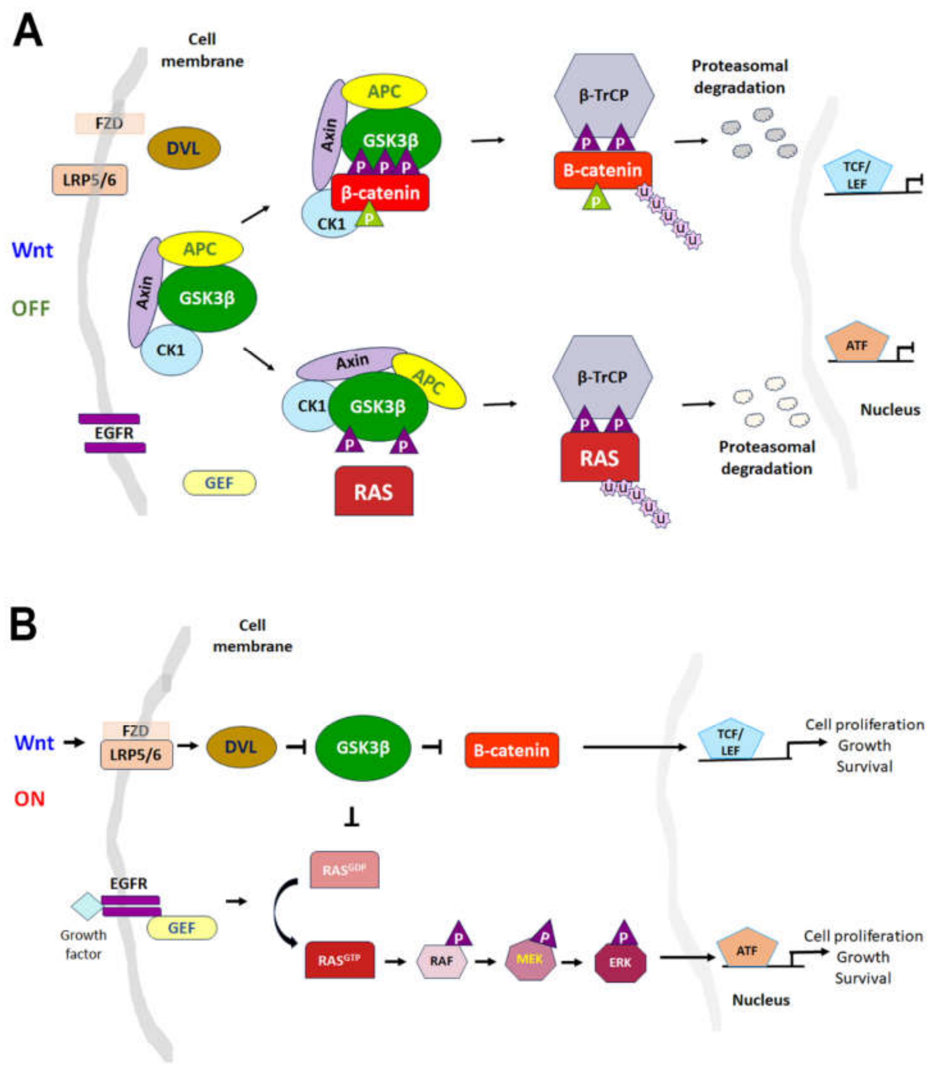

Figure S3: Diagrammatic presentation of crosstalk between Wnt and RAS signaling pathway. (A) The beta-catenin destruction complex (βCdC) consisting of APC, axin, GSK3β, and CK1 phosphorylates β-catenin with the help of GSK3β. This same protein complex (βCdC) also phosphorylates RAS protein by GSK3β. Next, phosphorylated β-catenin and RAS protein eventually leads to proteasomal degradation associated with the E3 ubiquitin ligase protein, β-TrCP. (B) In the presence of Wnt ligands, this βCdC dissociates itself from the degradation mechanism of β-catenin and RAS proteins and is associated with the plasma membrane-bound complex LRP5/6, FZD, and DVL. Freed β-catenin eventually enters the nucleus and influences various neoplasmic factors. When the β-TrCP does not degrade RAS, it is activated by the EGFR-associated GEF complex and shifts to RAS-GTP form. This eventually phosphorylates the ERK. Activated ERK enters the nucleus and is responsible for the cell's proliferation, growth, and survival.
